# Application of Balanced Scorecard in the Evaluation of a Complex Health System Intervention: 12 Months Post Intervention Findings from the BHOMA Intervention: A Cluster Randomised Trial in Zambia

**DOI:** 10.1371/journal.pone.0093977

**Published:** 2014-04-21

**Authors:** Wilbroad Mutale, Jeffrey Stringer, Namwinga Chintu, Roma Chilengi, Margaret Tembo Mwanamwenge, Nkatya Kasese, Dina Balabanova, Neil Spicer, James Lewis, Helen Ayles

**Affiliations:** 1 Department of Public Health, University of Zambia School of Medicine, Lusaka, Zambia; 2 Clinical Research Department, Faculty of Infectious and Tropical Diseases, London School of Hygiene and Tropical Medicine, London, United Kingdom; 3 Centre for Infectious Disease control in Zambia, Northmead Lusaka, Zambia; 4 ZAMBART Project, Ridgeway Campus, University of Zambia, Lusaka, Zambia; 5 Department of Global Health and Development, Faculty of Public Health and Policy, London School of Hygiene and Tropical Medicine, London, United Kingdom; 6 Department of Infectious Disease Epidemiology, Faculty of Epidemiology and Population Health, London School of Hygiene and Tropical Medicine, London, United Kingdom; University of Alabama at Birmingham, United States of America

## Abstract

**Introduction:**

In many low income countries, the delivery of quality health services is hampered by health system-wide barriers which are often interlinked, however empirical evidence on how to assess the level and scope of these barriers is scarce. A balanced scorecard is a tool that allows for wider analysis of domains that are deemed important in achieving the overall vision of the health system. We present the quantitative results of the 12 months follow-up study applying the balanced scorecard approach in the BHOMA intervention with the aim of demonstrating the utility of the balanced scorecard in evaluating multiple building blocks in a trial setting.

**Methods:**

The BHOMA is a cluster randomised trial that aims to strengthen the health system in three rural districts in Zambia. The intervention aims to improve clinical care quality by implementing practical tools that establish clear clinical care standards through intensive clinic implementations. This paper reports the findings of the follow-up health facility survey that was conducted after 12 months of intervention implementation. Comparisons were made between those facilities in the intervention and control sites. STATA version 12 was used for analysis.

**Results:**

The study found significant mean differences between intervention(I) and control (C) sites in the following domains: Training domain (Mean I:C; 87.5.vs 61.1, mean difference 23.3, p = 0.031), adult clinical observation domain (mean I:C; 73.3 vs.58.0, mean difference 10.9, p = 0.02 ) and health information domain (mean I:C; 63.6 vs.56.1, mean difference 6.8, p = 0.01. There was no gender differences in adult service satisfaction. Governance and motivation scores did not differ between control and intervention sites.

**Conclusion:**

This study demonstrates the utility of the balanced scorecard in assessing multiple elements of the health system. Using system wide approaches and triangulating data collection methods seems to be key to successful evaluation of such complex health intervention.

**Trial number:**

ClinicalTrials.gov NCT01942278

## Introduction

In many low income countries, delivery of quality health services is hampered by system wide barriers which are often interlinked and their contribution to outcomes difficult to establish [1], [2]. It is therefore important that health managers and researchers recognise this and use methods and approaches which take into account the complexity and connectedness across health system building blocks [1], [3], [4]. Some researchers have argued that part of the problem with the health systems debate and research is that it tends to adopt a reductionist perspective that ignores the complexity of the health system [5]. There are now calls for a paradigm shift in the way interventions are designed and evaluated [1]. Emphasis should be paid not only to outcomes but also to the processes leading to the observed outcomes [1], [6]. It has been recognised that taking a more comprehensive view that expands and challenges the status quo is more likely to provide lessons on what works and why[2], [7]–[9]. However, despite these recent advances in thinking around health systems, there are very few cases of studies empirically addressing these complexities in their design and interpretation of findings. A recent systematic review showed that many evaluations of complex interventions are too narrow and lack a system wide approach [10].

An approach such as a balanced scorecard allows for a comprehensive analysis of domains that are deemed important in achieving the overall vision of the health system [11], [12]. A balanced scorecard is a strategic management tool that was first suggested by Robert Kaplan and David Norton in 1992 [13]. It provides information on areas of strategic importance to guide future planning, but also serves as a snapshot of how well an organisation or system is performing [14]. It is made up of domains and indicators derived from the strategic vision of an organisation aimed at measuring its performance [15], [16].

Although the use of balanced scorecard in health care is being advocated, its application has been mostly limited to high income countries [13], [16]–[20]. The World Health Organisation has recently recommended the use of balanced scorecard in monitoring and evaluation of the health system building blocks [21]. Studies that have applied balanced scorecard have given arguments for adopting balanced scorecard approach in evaluating health system interventions and demonstrating that such a methodology has the potential to guide investments aimed at improving health system especially in low income countries [20]–[24]. The advantage with using a balanced scorecard is that it enables the focus on the overall vision while looking at the processes which are deemed important in achieving the overall goal [13], [15]. Crucially, the balanced scorecard approach provides means for researchers and health system managers to evaluate complex interventions [11].

Edward et al.2011, modified the original balanced scorecard making it more applicable in low income country health care settings. They highlighted six important domains for measuring health system strengthening [22]. Work done in Bangladesh by Khan et al.2012 has highlighted the central role that balance scorecard approaches could play in identifying barriers and facilitators of health system interventions and how data collection guided by balanced scorecard at health facility level could improve decision making [18]. In our recent publication, we applied the balanced scorecard approach to describe the baseline status of three BHOMA intervention districts in Zambia [25]. We reported the applicability of the balanced scorecard in the Zambian health care settings and the implication for evaluating health system interventions targeting the Millennium Development Goals. In this paper we extend this work by presenting preliminary findings after 12 months of implementation of the BHOMA intervention. The BHOMA study is a cluster randomised stepped wedge study of interventions aiming to strengthen the health system in three rural districts of Zambia. The evaluation of the BHOMA intervention utilises both qualitative and quantitative approaches. In this paper, we present the quantitative results of the follow-up study applying the balanced scorecard approach as described at baseline [25]. Qualitative results are presented elsewhere [26].

This study seeks to contribute to the generation of empirical evidence in health system research by utilising an innovative approach that offers an opportunity to assess multiple domains that exits in complex health systems.

## Methodology

The BHOMA study is a cluster randomised community intervention that aims to strengthen the health system in three rural districts covering 42 health facilities in Zambia with a total population of 306,000.

The study has a stepped wedge design where the intervention is being rolled-out gradually until all the 42 health facilities receive the intervention. The unit of randomisation is the health facility and its catchment population. The study has an integrated package of interventions, at both health facility and community level. The impact of the interventions is being measured through an evaluation of the interventions using selected endpoints including Standardised Mortality Rate in the population less than 60 years and under-five mortality. The evaluation data is being collected through community and health facility surveys. This paper focuses on the results of the health facility survey conducted in 2012 when 24 clusters were in the intervention phase of the intervention and 18 in the control phase.

The protocol for this trial and supporting CONSORT checklist are available as supporting information; see Protocol S1 and Checklist S1.

### Intervention Design

The BHOMA intervention is part of the African health initiative which aims improve population health in five sub Saharan Africa [27]. The intervention commenced in April 2011 when the first set of health facilities received the intervention. All the health facilities are expected to receive the intervention my mid 2013. The final evaluation of the BHOMA intervention will be 2014. In order to ensure objective evaluation, the BHOMA study is made up of two independent teams. The implementation is being done by the Centre for Infectious Diseases Control in Zambia (CIDRZ) while the Zambia AIDS Related Tuberculosis (ZAMBART) is evaluating the project. The teams work closely with each other and the Ministry of Health at national and district level.

The BHOMA intervention is made up of three primary strategies designed to work at different levels of the health system. These are district, health facility and community strategies. The full methodology is described elsewhere [28], [29]. Following is a summary description of the three BHOMA strategies:

### The District

In each of the three districts, one Quality Improvement (QI) team is introduced that implements the intervention in target health facilities. The order of implementation was determined at randomisation and the QI teams follow this order when introducing intervention in target heath facilities. Each QI team consists of two nurses and one clinical officer. The teams work closely with the Ministry of Health.

### The Health Facility Intervention

The health facility-based intervention aims to improve clinical care quality by implementing practical tools that establish clear clinical care standards, providing essential resources to meet these standards and communicating standards through intensive clinic implementations. Each clinic generates self assessment reports that help identify areas of weakness for further improvement with support from the quality improvement team. Leadership training is provided to the health workers targeting governance, finance, supply chain and human resource management. Staffing support consists of lay workers trained as “Clinic Supporters.” These lay workers are trained to assume as many non-clinical duties as possible. These include registration of patients, filing, triaging, recording vital signs, fast tracking urgent cases and routing patients through services.

### The Community Intervention

The BHOMA project has engaged community health workers on a part time basis. They are trained in providing preventive services and tracking missed clinic appointments. They work in collaboration with community health units known as Neighbourhood Health Committees (NHCs) and Traditional Birth Attendants (TBAs). The community health workers are also being trained in capturing and recording local health data and sending it to health facilities via mobile phones or physically.

**Figure 1:**
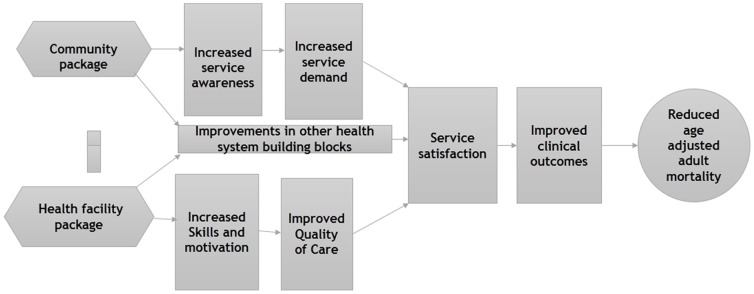
Summary of the BHOMA Intervention cascade.

Figure 1 gives a summary of the BHOMA intervention. The community strategy is expected to drive the demand for health services while the health facility strategy is expected to improve health worker skills, service quality and other health system building blocks. The overall effect of the intervention is to improve health outcomes.

### Sampling and Sample Size

**Figure 2:**
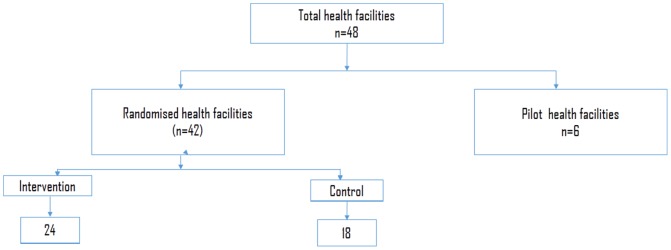
BHOMA intervention randomisation chart.

There were 48 eligible health facilities in the three BHOMA districts. Six were used for piloting the intervention and all the remaining 42 health facilities were included in the study (Figure 2). Sample size for community survey are reported elsewhere [28]. This paper is focusing on health facility surveys.

### Randomisation and Rollout Plans

**Figure 3:**
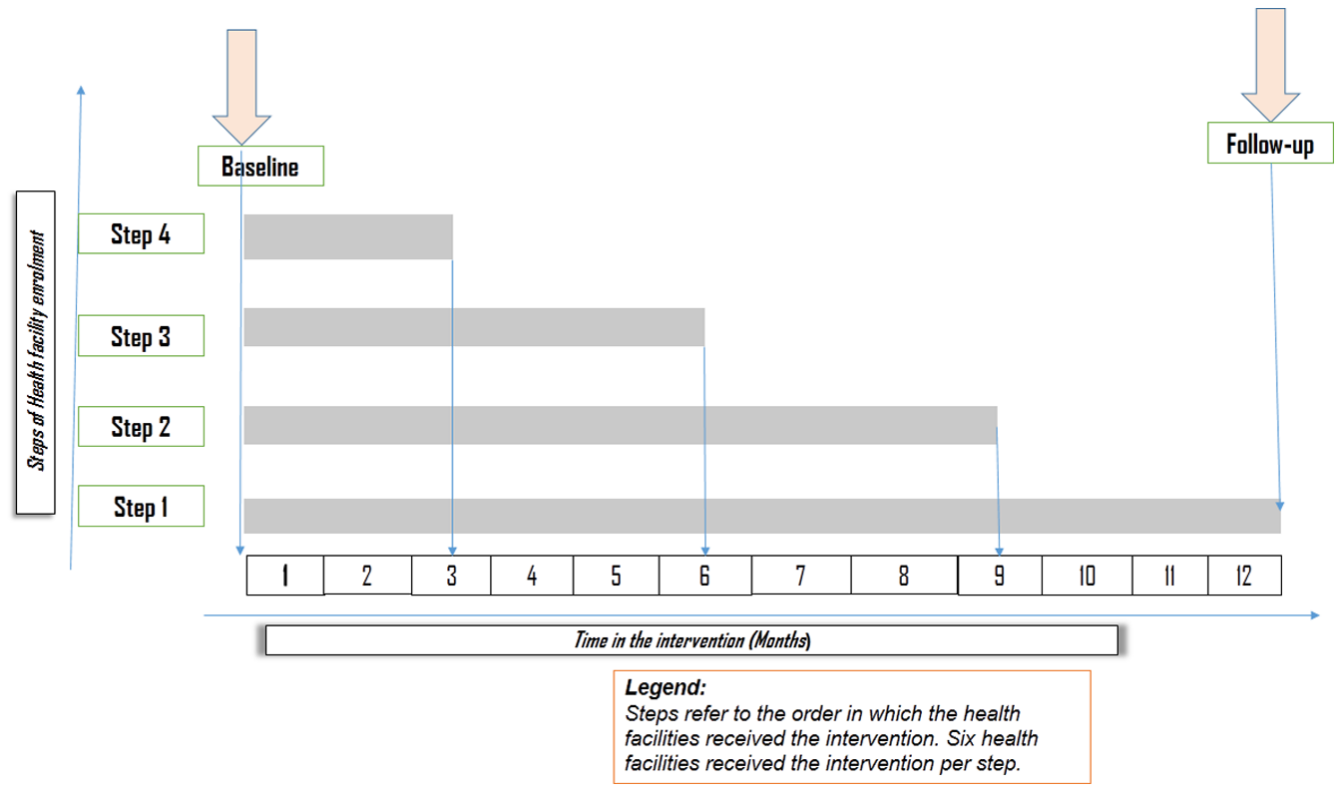
BHOMA intervention step wedged rollout over time.

The 42 health facilities were randomised in the order of receiving the intervention in a step wedge fashion until all receive the intervention. Six facilities are randomised to start the intervention in each step and each step took three months to implement. (Figure 3).

Randomisation was done by a statistician from London School of Hygiene and Tropical Medicine who had no prior knowledge of the study sites. Randomisation was stratified by district.

### Evaluation Design

#### Baseline survey

A baseline survey was conducted at the beginning of the intervention in 2011. A balanced scorecard was applied to rank the performance of the 42 target health facilities. The results of the baseline study have been reported elsewhere [25].

#### Follow-up study

A 12 month follow-up health facility survey was conducted in 42 health facilities between May and September, 2012. Appointments were made with managers before the research team visited each of the health facilities. At each health facility a number of questionnaires were administered targeting health facility managers, health workers and patients. All the study tools were interviewer administered except for the governance which was self administered. At each health facility the health facility officer in-charge and two other health workers were interviewed. Five observations of adult clinical encounters were done irrespective of the presenting complaint. Five observations of child clinical encounters were done with children being eligible if they were under five years and presenting with fever, cough or diarrhoea. Similarly five exit interviews for adults and five for under five child/guardian pair were done following the same approach described at baseline [25]. For specific tools and calculation of domain scores refer to Tools S1, S2, S3, S4, S5, S6, S7.

Data collection was conducted by the evaluating team composed of a team leader who is a medical doctor and epidemiologist, with 18 research assistants with a medical background. Data collectors were trained for five days on how to administer the study tools.

### Data Analysis

Data were double entered onto an Access database and exported to STATA version 12 for analysis. Simple frequencies were used to explore the data. Comparisons were made between intervention and control facilities stratified by district and the time in the intervention. We looked at effect of the intervention by time in the intervention to determine whether there was dose relationship. Linear regression was done to determine the correlations between measures of quality for children and adults with health system domains in the balanced scorecard [25]. We adjusted for cluster design using Stata version 12 estimation command with the vce(cluster clustvar) option to obtain a robust variance estimate that adjusts for within-cluster correlation [30]. We also adjusted for baseline scores, district and catchment population. Time in the intervention was left out of the model because of collinearity.

### Ethical Considerations

The study was approved by the University of Zambia Bioethics Committee and the London School of Hygiene and Tropical Medicine Ethics Committee. All participants were informed about the purpose of the survey and were asked to sign a consent form before taking part in the study. Parents/guardians signed consent forms on behalf their children. Those who could not write were asked to thumb print the consent form in the presence of an independent observer. Confidentiality was ensured during data collection and subsequent publication of the results.

## Results

### Health Facility Demographic Characteristics

In total there were 42 health facilities which were randomly allocated to the intervention or control. At the time of follow-up, 4 steps of the intervention had been implemented. 24 health facilities were in the intervention phase (I) while 18 had not received the intervention and so were in the control phase (C). For those health facilities that had received the intervention, 12 had been in the intervention phase for between 3–6 months and 12 for between 9–12 months. (See figure 3).

The majority of the health facilities were classified as rural (81% in Chongwe, 71% in Kafue and 57% in Luangwa). Two health facilities were part of mission hospitals (1 in Chongwe and 1 in Luangwa) neither of which had received the intervention. (See Table 1).

**Table 1:** Summary of health facilities in the intervention and control sites stratified by districts.

		Chongwe		Kafue		Luangwa	
	*Variable*	*n*	*%*	*n*	*%*	*n*	*%*
**Allocation:**	***Intervention***	12	57.1	8	57.1	4	57.1
	***Control***	9	42.9	6	42.9	3	42.9
**Total**		**21**	**100.0**	**14**	**100.0**	**7**	**100.0**
**Time intervention**:	***0***	9	50.0	6	50.0	3	50.0
	***3–6M***	6	33.3	4	33.3	2	33.3
	***9–12M***	6	16.7	4	16.7	2	16.7
**Total**		**18**	**100**	**14**	**100.0**	**7**	**100.0**
**Residence:**	***Rural***	17	81.0	10	71.4	4	57.1
	***Peri urban***	3	14.3	4	28.6	2	28.6
	Hospital*	1	4.8	0	0.0	1	14.3
**Total**		**21**	**100.0**	**14**	**100.0**	**7**	**100.0**

### Comparisons of Intervention and Control Health Facilities

Mean scores were calculated for each domain in the balanced scorecard and these are shown in table 2. The major differences in the mean scores between intervention(I) and control (C) health facilities were in the following domains: Training (mean I:C; 87.5.vs 61.1, mean difference 23.3, p = 0.031), adult clinical observation (mean I:C; 73.3 vs.58.0, mean difference 10.9, p = 0.02) and health information (mean I:C; 63.6 vs.56.1, mean difference 6.8, p = 0.003). These differences were statistically significant before and after adjusting for baseline score, catchment population and district. In addition to the above domains, infection control and tracer drugs showed statistically significant difference after adjusting for baseline score, catchment population and district. (i.e. infection control (mean I:C; 86 vs.78, mean difference 9.1, p = 0.03), Tracer drugs (mean I:C; 80 vs.77, mean difference 3.0, p = 0.05). Overall there was no gender differences in adult service satisfaction between control and intervention sites. In addition, governance and motivation scores did not differ between control and intervention sites.

**Table 2:** Balanced scorecard measure of the effect of the BHOMA intervention after 12 months of implementation stratified by district.

	Chongwe		Kafue		Luangwa		All Districts			
	*Intervention(n = 12)*	*Control (n = 9)*	*Intervention(8)*	*Control(6)*	*Intervention (4)*	*Control (3)*	*Intervention(24)*	*Control(18)*	Mean difference** *(95% CI)*	P-value
Domains A:Patients										
Patient satisfaction	69.2 (63.3–74.9)	73.3 (64.5–82.1)	70.7 (66.2–75.0)	69.4 (65.4–73.5)	75.8 (64.4–87.2)	65.6 (57.9–73.1)	70.7 (65.8–75.7)	70.7 (66.9–74.7)	1.4 (−4.5, 7.3)	0.64
Patient satisfaction	74.8 (68.4–81.1)	82.3 (76.8–87.9)	78.5 (73.7–83.3)	76.7 (72.9–80.4)	72.3 (71.1–73.4)	66.3 (56.7–75.9)	76.0 (71.9–79.3)	77.7 (73.4–82.2)	0.3–4.0−4.65	0.89
**Domain B: Human resources**										
Health worker motivation scores	77.0 (71.5–82.4)	79.4 (73.7–85.2)	71.6 (70.5–72.8)	73.8 (66.1–81.6)	83.0 (76.1–89.9)	77.4 (75.7–79.1)	76.3 (72.8–79.7)	77.2 (73.2–81.3)	−1.2 (−6.5–4.13)	0.60
Training domain	**100.0**	66.7* (34.5–98.8)	62.5 (38.6–97.7)	66.6 (35.6–97.8)	**100.0**	33.3* (−**22.3–88.9)**	87.5 (76.6–98.4	61.1* (39.0–83.2)	**23.3 (2.3–44.5)**	0.031*
**Domain C: Service capacity**										
Basic Infrastructure index	80.8 (73.9–87.6)	73.1 (64.5–81.6)	71.6 (60.9–82.3)	76.9 (67.6–86.1)	71.2 (65.3–77.1)	73.1 (69.4–76.8)	76.1 (70.7–81.5)	74.4 (68.9–79.7)	1.9 (−6.2–10.1)	0.63
Basic equipment index	75.8 (64.9–86.7)	87.2 (75.9–98.6)	73.0 (61.6–84.2)	73.3 (61.6–85.1)	82.6 (71.4–93.6)	74.0 (62.9–85.1)	75.8 (68.7–82.9)	80.4 (72.5–88.3)	−5.71 −15.7–4.3)	0.25
Laboratory capacity index	76.1 (69.4–82.8)	70.8 (58.4–83.4)	67.2 (54.3–80.1)	72.9 (57.4–88.5)	65.5 (48.4–82.9)	51.3 (34.6–67.9)	71.4 (64.9–77.9)	68.3 (58.9–77.6)	4.3 (−7.0–15.5)	0.44
Tracer drugs index	88.6 (85.6–91.6)	86.7 (84.2–89.6)	75.6 (72.3–78.8)	74.2 (70.7–77.8)	61.6 (57.3–65.4)	52.8 (50.5–55.2)	**79.7 (75.1–84.4)**	**76.9 (70.8–83.1)**	**3.0 (**−**0.0–6.1)**	**0.05**
Infection control index	89.3 (83.2–95.4)	84.1 (74.5–93.8)	82.3 (75.3–88.9)	76.2 (63.0–89.4)	82.1 (75.8–88.5)	61.4 (45.9–76.9)	**85.7 (81.5–89.9)**	**77.7 (69.7–85.7)**	**9.1 (0.9–17.2)**	**0.029**
**Domain D: Finance**										
Finance index	68.0 (60.6–75.5)	70.4 (63.2–77.5)	64.6 (53.4–75.8)	66.7 (66.7–67.6)	58.4 (49.8–66.9)	55.6 (46.3–64.9)	65.3 (59.6–70.9)	66.7 62.1–71.3)	−2.3 (−10.0–5.4)	0.54
**Domain E: Governance**										
Governance Index	77.3 (69.1–85.5)	81.0 (75.5–86.6)	85.3 (81.8–88.8)	83.3 (73.4–93.2)	87.9 (82.8–92.9)	85.8 (74.3–97.2)	81.7 (77.0–86.5)	82.6 (77.8–87.4)	−0.1 (−7.2–6.9)	0.98
**Domain F:Health information**										
Health information Index	66.2 (61.0–70.9)	57.8 (54.9–60.7)	60.8 (56.5–64.9)	55.7 (49.3–62.0)	**62.0 (53.6–70.4)**	56.0* (52.1–59.9)	**63.6 (60.2–66.9)**	**56.8 (54.1–59.5)**	**7.3 (2.6–12.0)**	0.003*
**Domain E: Service provision**										
Service readiness index	68.7 (59.1–70.1)	64.7 (59.1–70.2)	67.8 (62.3–73.2)	75.3 (70.6–80.1*)*	64.0 (57.9–69.6)	64.7 (62.3–73.2)	67.4 (63.9–70.9)	68.2 (64.1–72.2)	−0.3 (−5.5–4.9)	0.90
Clinical observation index(Children)	82.0 (63.3–99.99.9)	82.2 (59.6–104)	40.9 (22.6–59.2)	50.0 (34.0–65.9))	50.0 (16.1–83.9)	46.7 (17.2–76.1)	62.7 (48.1–77.5)	65.6 (49.9–81.2)	9.6 (−6.6–25.8)	
Clinical observation index(Adults)	63.7 (53.5–73.9)	53.3 (44.0–62.6	80.3 (74.4–86.1)	68.5 (57.3–79.7)	68.3 (63.4–73.2)	51.1 (46.8–55.4)	**70.3 (63.7–76.3)**	**58.0 (51.0–65.0)**	**10.9 (2.13–19.8)**	0.016*
Domain: Overall vision:										
Service satisfaction index by Gender:										
*Male*	77.8 (66.7–88.9)	78.2 (72.2–84.2)	75.6 (68.7–82.5)	75.4 (69.1–81.6)	74.0 (69.4–78.6)	73.7 (69.2–78.3)	76.6 (70.3–82.7)	78.6 (72.2–80.6)	−3.3 (−12.4–5.9)	0.47
*Female*	75.3 (69.1–81.6)	79.3 (75.6–82.9)	74.7 (70.6–78.8)	79.8 (74.5–85.2)	79.1 (71.6–86.6)	77.7 (65.9–89.5)	76.8 (72.0–79.6)	79.4 (72.2–80.6)	−2.7 (−8.3–2.8)	0.32

1*p<0.05.

2**mean difference adjusted for baseline score, catchment population and district.

### District Comparison of Intervention and Control Health Facilities

In Chongwe district, significant mean differences between intervention and control sites were reported in training domain (I:C; 100 vs.66.0.) and health information domain (mean I: C; 66.2 vs. 58.). Higher mean scores in the intervention were also noted in the Basic infrastructure domain (mean I: C; 81.0 vs.73), infection control domain (mean I: C; 89.3 vs.84.1) and adult clinical observations domain (mean I: C; 64.0 vs.53.0). However, the differences were not statistically significant.

In Kafue district, higher mean scores in the intervention were reported in infection control domain l (mean I:C; 82.2 vs.76.2), health information domain (mean I:C; (60.8 vs.55.7) and adult clinical observation domain (mean I:C; 80.3 vs.68.5). However, the differences were not statistically significant.

In Luangwa district, significant differences between Intervention and control sites were reported in the training domain (mean I: C; 100 vs.33.3) and infection control domain (82.1vs.61.4,) and adult clinical observation (mean I: C; 68.3 vs.51.1).

### Dose Dependence Effect of the Intervention

We compared the effect of the intervention by time in the intervention phase. Possible intervention dose effect was noted in the training domain which showed mean increase from 61.1 in the control to 87.5 when the intervention had been in place for 3–6 months and remained stable after the intervention had been in place for between 9–12 months. The adult clinical observations domain showed a similar trend rising from 58 in the control to 68 at 3–6 months and to 72 at 9–12 months of intervention time. These differences were statistically significant. (p<0.05). The domain for Basic equipment showed improvement soon after intervention but deteriorated with time (mean at 3–6 months 78 to 74 at 9–12 months). (See Table 3).

**Table 3:** Balanced scorecard measure of the effect of the BHOMA intervention after 12 months of implementation stratified by timing of roll ou.

Domain	Time in the intervention		
	Control n = 18	3–6 months n = 12	9–12 months n = 12
**Domain A: Patient and community**			
Patient satisfaction children index	70.7 (65.7–75.7)	69.7 (63.2–76.2)	71.8 (67.5–76.1)
Patient satisfaction Adult index	77.7 (73.4–82.2)	71.1 (66.1–76.0)	80.1 (76.1–84.1)
**Domain B: Human resources**			
Health worker motivation scores	77.2 (73.2–81.3)	75.7 (70.7–80.6)	76.8 (72.1–81.6)
Training in the past 12 months	**61.1 (39.0–83.2)**	**87.5** * **(69.9–105.1)**	**87.5** * **(74.7–100.3)**
**Domain C: Service capacity**			
Basic Infrastructure index	74.4 (68.9–79.7)	78.2 (72.2–84.2)	74.0 (65.2–82.9)
Basic equipment index	80.4 (72.5–88.3)	80.0 (71.4–88.5)	71.7 (60.9–82.5)
Laboratory capacity index	68.2 (58.9–77.6)	75.6 (69.6–81.5)	67.2 (56.2–78.2)
Tracer drugs index	76.9 (70.8–83.1)	80.6 (74.1–87.2)	78.8 (72.2–85.4)
Infection control index	77.7 (69.7–85.7)	89.3 (84.2–94.3)	82.1 (76.1–88.2)
**Domain D: Finance**			
Finance index	66.6 (62.1–71.3)	63.9 (58.4–69.3)	66.7 (56.8–76.5)
**Domain E: Governance**			
Governance Index	82.6 (77.8–87.4)	80.4 (74.0–86.8)	83.1 (76.2–90.0)
**Domain F:Health information**			
Health information Index	**56.8** * **(54.1–59.5)**	**63.5** * **(58.3–68.7)**	**63.7** * **(59.4–67.9)**
**Domain E: Service provision**			
Service readiness index	68.2 (64.2–72.2)	69.1 (63.2–74.9)	65.8 (62.2–69.2)
Clinical observation index (Children)	65.6 (49.8–81.2)	66.7 (46.6–86.7)	58.9 (37.6–80.2)
Clinical observation index (Adults)	58.0 (51.0–65.0)	68.3 (57.4–79.2)	71.7 (65.4–77.9)
**Domain: Overall vision:**			
Service satisfaction index by Gender:			
*Male*	76.4 (72.2–80.6)	73.7 (63.7–83.6)	80.0 (75.0–79.4)
*Female*	79.2 (75.9–82.6)	75.9 (72.4–79.4)	75.6 (69.4–82.1)

*P<0.05.

### Linear Regression Model

Linear regression was done with the following dependent variables: Adult Clinical observation and service satisfaction scores, Children clinical observation and service satisfaction scores. In addition to all the health system domains applied at baseline [25], an intervention variable was added to the model. The model was adjusted for baseline scores, catchment population and district. There was no difference in children clinical observation score between the intervention and controls sites. However, children clinical observation was significantly correlated with service readiness (coef 1.2, p = 0.01) and health worker motivation (coef 0.44, p = 0.09).

There was a statistically significant difference in adult clinical observation score between intervention and control sites (coef 23.29, p = 0.01). Other domains which correlated with adult clinical observation were; laboratory capacity (coef 0.25, p = 0.04), training (coef 0.16, p = 0.07 and health information (coef 0.87, p = 0.01). There was no difference in adult satisfaction score between the intervention and control sites. However, adult satisfaction score was correlated with health information, (coef 0.29, p = 0.02, service readiness (coef 0.34, p = 0.04), children clinical observation (coef 0.14, p = 0.08) and children satisfaction score (Coef 0.23, p = 0.07). (See Table 4).

**Table 4:** Linear regression analysis of the association between the different measures of quality of care.

	Model 1: Dependent variable: Children Clinical observation			Model 2 Dependent variable: Adult clinical observation			Model 3: Dependent variable: Children satisfaction score			Model 4: Dependent variable: Adult satisfaction score		
	Coef	Std err	p	Coef	Std err	p	Coef	Std err	p	Coef	Std err	p
**Intervention**	7.27	15.32	0.64	**23.29**	**5.09**	**0.01**	6.07	5.85	0.31	3.5	2.7	0.21
**Health worker motivation scores**	−0.46	0.79	0.56	**0.68**	**0.33**	**0.04**	**0.44**	**0.26**	**0.09**	−0.05	0.16	0.74
**Training**	0.16	0.13	0.26	**0.16**	**0.07**	**0.02**	0.04	0.05	0.37	−0.01	0.03	0.92
**Basic Infrastructure score**	0.08	0.29	0.77	−0.03	0.15	0.84	0.13	0.13	0.12	−0.16	0.09	0.09
**Basic equipment index**	0.08	0.26	0.74	0.16	0.14	0.29	−0.06	0.09	0.51	−0.01	0.08	0.83
**Laboratory capacity score**	−0.02	0.29	0.95	**0.25**	**0.11**	**0.04**	0.15	0.11	0.16	−0.04	0.06	0.56
**Tracer drugs score**	1.06	1.10	0.34	−0.18	0.65	0.78	0.18	0.43	0.68	−0.04	0.23	0.86
**Infection control score**	0.76	0.33	0.03	−0.08	0.19	0.67	−0.09	0.12	0.40	0.05	0.12	0.66
**Health information score**	0.25	0.61	0.69	**0.87**	**0.31**	**0.01**	0.37	0.22	0.11	**0.29**	**0.11**	**0.02**
**Governance score**	0.14	0.37	0.70	−0.29	0.35	0.42	0.09	0.15	0.56	0.02	0.10	0.82
**Finance score**	0.33	0.21	0.95	0.24	0.71	0.62	1.2	0.47	0.78	0.34	0.17	0.76
**Service readiness score**	**1.21**	**0.41**	**0.01**	0.48	0.31	0.13	0.29	0.20	0.32	**0.34**	**0.18**	**0.04**
**Rural residence**	−8.49	15.5	0.59	11.5	7.5	0.14	5.20	6.71	0.44	0.28	4.7	0.95
**Children Clinical observation score**	–	–	–	0.07	0.10	0.52	0.27	0.14	0.21	**0.05**	**0.03**	**0.07**
**Adult Clinical observation score**	0.08	0.41	0.86	–	–	–	−0.18	0.19	0.38	**0.14**	**0.08**	**0.08**
**Children satisfaction score**	−0.04	0.43	0.91	−0.29	0.35	0.42	–	–	–	**0.23**	**0.12**	**0.07**
**Adult satisfaction score**	**1.43**	**0.59**	**0.02**	0.56	0.35	0.11	0.49	0.29	0.10	–	–	–

## Discussion

This study aimed to apply innovative approaches in evaluating a complex health system intervention and hence contribute to generation of empirical evidence to guide health system strengthening investments [31]. Most of the current discussions in this area are at the level of framework or theory but there is lack of empirical data especially from low income countries [3], [32], [33]. Applying a system wide approach in the form of balanced scorecard allowed for a comprehensive analysis of the different domains of the health system and how each was affected by the intervention [10], [34].

The results showed that the BHOMA intervention led to improvements in some domains of the balanced scorecard while other domains remained unaffected. Significant differences between intervention and control sites were only seen in adult clinical observation, training and health information domains. These differences remained significant when analysis was stratified by district. We acknowledge that these results are still interim as our follow up time ranged between 3 and 12 months only, with the last step of health facilities having the intervention for just 3 months. Nonetheless, the results point to some positive effect of the BHOMA intervention regardless of the study district, time in the intervention or baseline scores. We will be able to assess the full effect of the BHOMA intervention when the final assessment is made in 2014.

Interestingly, some domains such as health worker motivation, service satisfaction for children and adults and governance did not show differences between intervention and control sites despite the presence of the BHOMA intervention. This remained true even after adjusting for baseline scores and showed no evidence of dose dependence. It remains unclear why these domains did not respond to the intervention but the short observation time could partly explain this. Complex system theory acknowledges delays between cause and effect [35]. It will be interesting to see how these domains respond with longer intervention time. Other possible explanations have been explored in the qualitative component of the BHOMA evaluation reported elsewhere [26].

Linear regression analysis showed that adult clinical observation score was one measure of service quality that showed statistically significant differences between control and intervention sites. This might be a more sensitive marker of the effect of the intervention which could be useful when evaluating similar interventions aimed at strengthening complex health systems. The children measures of quality did not show any significant difference between intervention and control even after adjusting for catchment population and baseline scores. We reported at baseline that the children measures of quality had lower scores when compared to adults [25]. The current results suggest that child services might still be lagging behind adult services in the BHOMA intervention. This was attributed to low number of health workers being trained in the integrated management of childhood illnesses (IMCI) in most study sites. However, we also acknowledge the limitation reported by other studies done in low income settings which have shown that in-service training may not necessarily translate in behaviour change that support quality improvements [36]. This might be the case in some of the domains that failed to show differences in the presence of intervention, although further follow up is required to confirm this.

Another lesson being learnt from the evaluation was that the effect of the intervention needs to be considered with contextual factors [37], [38]. These were noted to positively or negatively affect the intervention. In our study, we noted that health facilities located in peri-urban areas with larger catchment population and high patient volume seem to perform poorly in most domains despite the presence of the intervention. Their poor performance generally affected the scores across most domains in the intervention sites as all the bigger health facilities had received the intervention. This observation was important as the effect of the intervention could not be guaranteed by simple randomisation but that context was a critical determinant of how well the site performed in the presence of the intervention. Detailed analysis of individual health facilities revealed that hospital based health facilities strongly confounded the mean scores in the control sites as none had received the intervention but still scored very highly in most domains at baseline [25] and follow-up even in the absence of the intervention. In recent times context has been recognised as an important factor that could affect even well designed clinical trials and currently there are efforts to standardise collection of contextual information in clinical trials. Our findings agree with these observations and support efforts to have contextual data considered in understanding the mechanism of change in trial settings [39], [40].

The study had a number of limitations that must be considered when interpreting our findings. Firstly, the study was not powered to look for differences between sites or different types of facilities and therefore we will need to wait for final evaluation before any further interpretation of these findings. Secondly, the time from the implementation to the timing of this interim analysis was relative short. The longest intervention step had received the intervention for 12 months while the last step had received the intervention for 3 months only. This makes the comparison between control and intervention more complex requiring cautious interpretation. We have tried to explore the effect of the intervention by time or step. However, the results remained inconclusive. It is therefore recommended to see the end line evaluation that includes a community survey to make concrete conclusions about the effect of the intervention.

Some study results were based on observation of health workers and how they performed their duties in clinical setting. The fact that they were under observation could have altered their usual behaviour positively or negatively depending on what might be desirebale [41], hence biasing the results of our study. Similarly, exit interviews with clients could be influenced by this form of information bias [42].

The study was done mainly in rural districts of Zambia were health system challenges might be different from urban settings. Therefore our findings could be more applicable to similar rural settings and may not be generalised to urban settings. In addition, the study sites were fixed and limited to 42 health facilities based on what was available in the selected districts. This resulted in small sample size especially when performing stratified analysis by districts. This was worse in Luangwa district which had only 7 health facilities.

### Conclusion

This preliminary results show that the balanced scorecard approach can be useful in assessing the effects of complex public health interventions. In evaluation of complex interventions such as the BHOMA, attention should be paid to context. Using system wide approaches and triangulating data collection methods seems to be key to successful evaluation of such complex intervention.

## Supporting Information

Tools S1 **Calculation of health facility scores.**
(DOC)

Tools S2 **Adult Clinical observation checklist.**
(DOC)

Tools S3 **Children clinical observation checklist.**
(DOC)

Tools S4 **Finance tool.**
(DOC)

Tools S5 **Governance tool.**
(DOC)

Tools S6 **Health worker motivation tool.**
(DOC)

Tools S7 **Service satisfaction for adults and children.**
(DOC)

Checklist S1 **Consort for cluster randomised Trial.**
(DOCX)

Protocol S1 **Published protocol.**
(PDF)

## References

[1] Swanson RC, Cattaneo A, Bradley E, Chunharas S, Atun R, et al (2012) Rethinking health systems strengthening: key systems thinking tools and strategies for transformational change. Health Policy Plan 27 Suppl 4iv54–61. 23014154 10.1093/heapol/czs090 PMC3529625

[2] English M, Nzinga J, Mbindyo P, Ayieko P, Irimu G, et al (2011) Explaining the effects of a multifaceted intervention to improve inpatient care in rural Kenyan hospitals–interpretation based on retrospective examination of data from participant observation, quantitative and qualitative studies. Implement Sci 6: 124. 22132875 10.1186/1748-5908-6-124 PMC3248845

[3] Adam T, De Savigny D (2012) Systems thinking for strengthening health systems in LMICs: Need for a paradigm shift. Health Policy and Planning 27: iv1–iv3. 23014149 10.1093/heapol/czs084

[4] Agyepong IA, Ko A, Adjei S, Adam T (2012) When “Solution of yesterday become problems of today”Crisis-ridden decision maling in a complex adaptive sytem(CAS)-the additional Duty Hours Allowance in Ghana. Health Policy 27. 10.1093/heapol/czs083 23014150

[5] Julio Frenk (2010) The Global Health System: Strengthening National Health Systems as the Next Step for Global Progress. PlosMedicine 7. 10.1371/journal.pmed.1000089 PMC2797599 20069038

[6] English M, Schellenberg J, Todd J (2011) Assessing health system interventions: key points when considering the value of randomization. Bull World Health Organ 89: 907–912. 22271948 10.2471/BLT.11.089524 PMC3260899

[7] Paina L, Peters DH (2012) Understanding pathways for scaling up health services through the lens of complex adaptive systems. Health Policy Plan 27: 365–373. 21821667 10.1093/heapol/czr054

[8] Targreed A, Hsu J, de Savigny D, Lavis NJ, Rottingen JA, Bennet S, (2012) Evalauting health systems strengthening Interventions in low-income countries:Are we asking the right questions? Health Policy 27. 10.1093/heapol/czs086 23014156

[9] Targreed A, Hsu J, de Savigny D (2012) Systems thinking for strengthening health systems in LMICS:need for a paradigm shift. Health Policy 27. 10.1093/heapol/czs084 23014149

[10] Taghreed A, Hsu J, de Savigny D, Lavis NJ, Rottingen JA, Bennet S, (2012) Evalauting health systems strengthening Interventions in low-income countries:Are we asking the right questions? Health Policy 27. 10.1093/heapol/czs086 23014156

[11] Yap C, Siu E, Baker GR, Brown AD (2005) A comparison of systemwide and hospital-specific performance measurement tools. Journal of Health Care Management 50: 4. 16130808

[12] Kunz H, Schaaf T (2011) General and specific formalization approach for a Balanced Scorecard: An expert system with application in health care. Expert Systems with Applications 38: 1947–1955.

[13] Kaplan RS, Norton DP (1992) The balanced scorecard–measures that drive performance. Harv Bus Rev 70: 71–79. 10119714

[14] Gauld R, Al-wahaibi S, Chisholm J, Crabbe R, Kwon B, et al (2011) Scorecards for health system performance assessment: the New Zealand example. Health Policy 103: 200–208. 21723641 10.1016/j.healthpol.2011.05.016

[15] Lupi S, Verzola A, Carandina G, Salani M, Antonioli P, et al (2011) Multidimensional evaluation of performance with experimental application of balanced scorecard: a two year experience. Cost Eff Resour Alloc 9: 7. 21586111 10.1186/1478-7547-9-7 PMC3118336

[16] Inamdar SN, Kaplan RS, Jones ML, Menitoff R (2000) The Balanced Scorecard: a strategic management system for multi-sector collaboration and strategy implementation. Qual Manag Health Care 8: 21–39. 10.1097/00019514-200008040-00004 11183582

[17] Bisbe J, Barrubes J (2012) The Balanced Scorecard as a Management Tool for Assessing and Monitoring Strategy Implementation in Health Care Organizations. Rev Esp Cardiol S0300–8932(12)00383–1 [pii]/j.recesp.2012.05.014 [doi]. 10.1016/j.recesp.2012.05.014 22917775

[19] Khan MM, Hotchkiss D, Dmytraczenko T, Zunaid Ahsan K (2012) Use of a Balanced Scorecard in strengthening health systems in developing countries: an analysis based on nationally representative Bangladesh Health Facility Survey. Int J Health Plann Manage 10.1002/hpm.2136 [doi]. 10.1002/hpm.2136 22887590

[20] Inamdar N, Kaplan RS, Bower M (2002) Applying the balanced scorecard in healthcare provider organizations. J Healthc Manag 47: 179–195 discussion 195–176. 12055900

[21] Jeffs L, Merkley J, Richardson S, Eli J, McAllister M (2011) Using a nursing balanced scorecard approach to measure and optimize nursing performance. Nurs Leadersh (Tor Ont) 24: 47–58. 21512337 10.12927/cjnl.2011.22334

[22] WHO (2010) Monitoring the Building Blocks of healh systems: A handbook of Indiactors and their measurement stragegies.

[23] Edward A, Kumar B, Kakar F, Salehi AS, Burnham G, et al (2011) Configuring balanced scorecards for measuring health system performance: evidence from 5 years’ evaluation in Afghanistan. PLoS Med 8: e1001066. 21814499 10.1371/journal.pmed.1001066 PMC3144209

[24] El-Jardali F, Saleh S, Ataya N, Jamal D (2011) Design, implementation and scaling up of the balanced scorecard for hospitals in Lebanon: policy coherence and application lessons for low and middle income countries. Health Policy 103: 305–314. 21658787 10.1016/j.healthpol.2011.05.006

[25] Bouland DL, Fink E, Fontanesi J (2011) Introduction of the Balanced Scorecard into an academic department of medicine: creating a road map to success. J Med Pract Manage 26: 331–335. 21815545

[26] Mutale W, Godfrey-Fausset P, Mwanamwenge MT, Kasese N, Chintu N, et al (2013) Measuring health system strengthening: application of the balanced scorecard approach to rank the baseline performance of three rural districts in Zambia. PLoS One 8: e58650. 23555590 10.1371/journal.pone.0058650 PMC3605425

[27] Mutale W, Chintu N, Stringer JS, Chilengi R, Mwanamwenge MT, et al.. (2013) Application of system thinking: Evaluation of the BHOMA health system strengthening intervention in Zambia:.

[28] Sherr K, Requejo J, Basinga P (2013) Implementation research to catalyze advances in health systems strengthening in sub-Saharan Africa: the African Health Initiative. BMC Health Services Research 13: S1. 10.1186/1472-6963-13-S2-S1 PMC3668282 23819761

[29] Stringer J, Chisembele-Taylor A, Chibwesha C, Chi H, Ayles H, et al (2013) Protocol-driven primary care and community linkages to improve population health in rural Zambia: the Better Health Outcomes through Mentoring and Assessment (BHOMA) project. BMC Health Services Research 13: S7. 10.1186/1472-6963-13-S2-S7 PMC3668289 23819614

[30] Mutale W, Mwanamwenge TM, Chintu N, Stringer J, Balabanova D, et al.. (2013) Application of system thinking concepts in health system strengthening in low income settings: A proposed conceptual framework for the evaluation of a complex health system intervention: The case of the BHOMA intervention in Zambia:. Lusaka: ZAMBART. 10.1111/jep.12160 24814988

[32] Jeffrey M Wooldridge (2010) Econometric Analysis of Cross Section and Panel Data, Second Edition: MIT Press.

[33] Adam T, Hsu J, De Savigny D, Lavis JN, Rottingen JA, et al (2012) Evaluating health systems strengthening interventions in low-income and middle-income countries: Are we asking the right questions? Health Policy and Planning 27: iv9–iv19. 23014156 10.1093/heapol/czs086

[34] WHO (2009) Systems Thinking for Health Systems Strengthening.

[35] Atun R (2012) Health systems, systems thinking and innovation. Health Policy and Planning 27: iv4–iv8. 23014152 10.1093/heapol/czs088

[36] Taghreed A, Hsu J, de Savigny D (2012) Systems thinking for strengthening health systems in LMICS:need for a paradigm shift. Health Policy 27. 10.1093/heapol/czs084 23014149

[37] BeLue R, Carmack C, Myers KR, Weinreb-Welch L, Lengerich EJ (2012) Systems thinking tools as applied to community-based participatory research: a case study. Health Educ Behav 39: 745–751. 22467637 10.1177/1090198111430708

[38] Wasunna B, Zurovac D, Bruce J, Jones C, Webster J, et al (2010) Health worker performance in the management of paediatric fevers following in-service training and exposure to job aids in Kenya. Malar J 9: 261. 20849650 10.1186/1475-2875-9-261 PMC2955674

[39] Byrne A, Morgan A, Soto EJ, Dettrick Z (2012) Context-specific, evidence-based planning for scale-up of family planning services to increase progress to MDG 5: health systems research. Reprod Health 9: 27. 23140196 10.1186/1742-4755-9-27 PMC3563623

[40] de Savigny D, Webster J, Agyepong IA, Mwita A, Bart-Plange C, et al (2012) Introducing vouchers for malaria prevention in Ghana and Tanzania: context and adoption of innovation in health systems. Health Policy Plan 27 Suppl 4iv32–43. 23014151 10.1093/heapol/czs087

[41] Grant A, Treweek S, Dreischulte T, Foy R, Guthrie B (2013) Process evaluations for cluster-randomised trials of complex interventions: a proposed framework for design and reporting. Trials 14: 15. 23311722 10.1186/1745-6215-14-15 PMC3600672

[42] English M (2013) Designing a theory-informed, contextually appropriate intervention strategy to improve delivery of paediatric services in Kenyan hospitals. Implementation Science 8. 10.1186/1748-5908-8-39 PMC3620707 23537192

[43] Horwitz O, Lysgaard-Hansen B (1975) Medical observations and bias. Am J Epidemiol 101: 391–399. 1130402 10.1093/oxfordjournals.aje.a112107

[44] Van Ryckeghem DM, Crombez G, Goubert L, De Houwer J, Onraedt T, et al (2013) The predictive value of attentional bias towards pain-related information in chronic pain patients: a diary study. Pain 154: 468–475. 23375161 10.1016/j.pain.2012.12.008

