# Supplementary material for: Application of Balanced Scorecard in the Evaluation of a Complex Health System Intervention: 12 Months Post Intervention Findings from the BHOMA Intervention: A Cluster Randomised Trial in Zambia
Source: PLoS One. 2014 Apr 21;9(4):e93977. doi: 10.1371/journal.pone.0093977 (PMC3994016; doi:10.1371/journal.pone.0093977)
Supplement: Tools S1 — Calculation of health facility scores. (DOC) [file pone.0093977.s001.doc]

| | **INDICATOR** | | | | --- | --- | --- | | **Basic Infrastructure** | | | | 1. **The health facility at least one overnight or inpatient bed** | No | 0 | | Yes | 1 | |  | | |
| --- | --- | --- | --- | --- | --- | --- | --- | --- | --- | --- | --- | --- | --- |
| |  | **Is there 24 hour staff coverage?** | | | | --- | --- | --- | --- | |  | | Yes, 24 hour duty roster observed or staff live on site | 1 | |  | | Yes, 24 hour coverage but no duty roster and no staff living on site | 0.5 | |  | | No duty roster observed or no 24 hour coverage | 0 | |
| |  | **The facility has at least one qualified health worker** | | | | --- | --- | --- | --- | |  |  | No | 0 | |  |  | Yes | 1 | |
| |  | **The facility has at least one in the outpatient clinic area where both auditory and visual privacy can be maintained for client only has inpatients, services** | | | | --- | --- | --- | --- | |  |  | No | 0 | |  |  | Yes | 1 | |
| |  | **The facility has a working phone or shortwave radio to call outside that is available at all times** | | | | --- | --- | --- | --- | |  |  | No | 0 | |  | Only pay phone or personal cell phone | 0.5 | |  |  | Yes available on site | 1 | |
| |  | **The facility has access to a functional ambulance or other vehicle for emergency transportation for clients** | | | | --- | --- | --- | --- | |  |  | No | 0 | |  |  | Yes | 1 | |
| |  | **The facility has access to power (Electricity or Solar)** | | | | --- | --- | --- | --- | |  |  | No | 0 | |  |  | Yes | 1 | |  | **The power source is working today** | | | |  |  | No | 0 | |  |  | Yes |  | |
| |  | **The facility has a back up or stand-by generator for electricity** | | | | --- | --- | --- | --- | |  |  | No | 0 | |  |  | Yes | 1 | |
| |  | | **The Facility has access to safe water** | | | | --- | --- | --- | --- | --- | |  | No | | 0 | | |  | Yes | | 1 | | |  | |  | |  | |
| | 1. . **Water is water available on the day of survey**   0  No    1  Yes | | --- | |
| |  | | **The facility has a toilet (latrine) that is available for clients to use** | | | --- | --- | --- | --- | |  | No | | 0 | |  | Yes | | 1 | |
| |  | **Condition of the toilet/latrine** | | | | --- | --- | --- | --- | |  |  | Not Functioning | 0 | |  |  | Functioning | 1 | |
| |  | | | |  | | --- | --- | --- | --- | --- | | **Service availability** | | **No** | **Yes** | | |  | Immunisation services for children and child health | 0 | 1 | | |  | Family planning | 0 | 1 | | |  | Antenatal care | 0 | 1 | | |  | Any normal delivery services | 0 | 1 | | |  | PMTCT services (HIV test and ARV) | 0 | 1 | | |  | Treatment for STIS | 0 | 1 | | |  | Treatment or client follow up for TB | 0 | 1 | | |  | Voluntary counselling and testing (client walk-in) | 0 | 1 | | |  | Palliative treatment services (OI or pain) for AIDS | 0 | 1 | | |  | ART treatment or client follow up. | 0 | 1 | | |
| | **Service guidelines** | | **Observed** | **Reported but not seen** | **Not available** | | --- | --- | --- | --- | --- | |  | Immunisation services for children and child health | 1 | 0.5 | 0 | |  | Family planning | 1 | 0.5 | 0 | |  | Antenatal care | 1 | 0.5 | 0 | |  | Any normal delivery services | 1 | 0.5 | 0 | |  | PMTCT services (HIV test and ARV) | 1 | 0.5 | 0 | |  | Treatment for STIS | 1 | 0.5 | 0 | |  | Treatment or client follow up for TB | 1 | 0.5 | 0 | |  | Voluntary counselling and testing (client walk-in) | 1 | 0.5 | 0 | |  | Palliative treatment services (OI or pain) for AIDS | 1 | 0.5 | 0 | |  | ART treatment or client follow up. | 1 | 0.5 | 0 | |

| | 1. **Register with minimum information**   **Observed** | | **Reported but not seen** | **Not available** |  | | | --- | --- | --- | --- | --- | --- | |  | Immunisation services for children and child health | 1 | 0.5 | 0 | | |  | Family planning | 1 | 0.5 | 0 | | |  | Antenatal care | 1 | 0.5 | 0 | | |  | Any normal delivery services | 1 | 0.5 | 0 | | |  | PMTCT services (HIV test and ARV) | 1 | 0.5 | 0 | | |  | Treatment for STIS | 1 | 0.5 | 0 | | |  | Treatment or client follow up for TB | 1 | 0.5 | 0 | | |  | Voluntary counselling and testing (client walk-in) | 1 | 0.5 | 0 | | |  | Palliative treatment services (OI or pain) for AIDS | 1 | 0.5 | 0 | | |  | ART treatment or client follow up. | 1 | 0.5 | | 0 | |
| --- | --- | --- | --- | --- | --- | --- | --- | --- | --- | --- | --- | --- | --- | --- | --- | --- | --- | --- | --- | --- | --- | --- | --- | --- | --- | --- | --- | --- | --- | --- | --- | --- | --- | --- | --- | --- | --- | --- | --- | --- | --- | --- | --- | --- | --- | --- | --- | --- | --- | --- | --- | --- | --- | --- | --- | --- | --- | --- | --- | --- | --- | --- | --- | --- | --- | --- |
| | **Register last updated** | | Within last 7 days | | More than 7 days ago | |  | | --- | --- | --- | --- | --- | --- | --- | |  | Immunisation services for children and child health | | 1 | | 0 | | |  | Family planning | | 1 | | 0 | | |  | Antenatal care | | 1 | | 0 | | |  | Any normal delivery services | | 1 | | 0 | | |  | PMTCT services (HIV test and ARV) | | 1 | | 0 | | |  | Treatment for STIS | | 1 | | 0 | | |  | Treatment or client follow up for TB | | 1 | | 0 | | |  | Voluntary counselling and testing (client walk-in) | | 1 | | 0 | | |  | Palliative treatment services (OI or pain) for AIDS | | 1 | | 0 | | |  | ART treatment or client follow up. | | 1 | | 0 | | |
| | **Staff trained in last 12 months** | | **No** | **Yes** | | --- | --- | --- | --- | |  | Immunisation services for children and child health | 0 | 1 | |  | Family planning | 0 | 1 | |  | Antenatal care | 0 | 1 | |  | Any normal delivery services | 0 | 1 | |  | PMTCT services (HIV test and ARV) | 0 | 1 | |  | Treatment for STIS | 0 | 1 | |  | Treatment or client follow up for TB | 0 | 1 | |  | Voluntary counselling and testing (client walk-in) | 0 | 1 | |  | Palliative treatment services (OI or pain) for AIDS | 0 | 1 | |  | ART treatment or client follow up. | 0 | 1 | |
|  |
| | **Basic Equipment** | | --- | |
| |  | **Equipment Availability** | **Observed and functioning** | **Observed but not functioning** | **Not available** | | --- | --- | --- | --- | --- | |  | Autoclave | 1 | 0.5 | 0 | |  | Adult weighing scale | 1 | 0.5 | 0 | |  | Infant weighing scale (gradations at minimum 100 gm) | 1 | 0.5 | 0 | |  | Thermometer | 1 | 0.5 | 0 | |  | Stethoscope | 1 | 0.5 | 0 | |  | Blood Pressure Cuff | 1 | 0.5 | 0 | |  | Suction Tube | 1 | 0.5 | 0 | |  | Suction machine | 1 | 0.5 | 0 | |  | Mask/Ambu bag | 1 | 0.5 | 0 | |  | Timer/Watch | 1 | 0.5 | 0 | |
|  |
| **Laboratory capacity** |
| |  | | | | | | --- | --- | --- | --- | --- | |  |  |  | |  | |  |  | **Test can be conducted onsite today** | **Observed system for test outside, receive results back** | **Test not available** | |  | Full Blood Count | 1 | 0.5 | 0 | |  | Anaemia (Haemoglobin, Haematocrit or litmus paper | 1 | 0.5 | 0 | |  | Malaria (rapid test or microscopy) | 1 | 0.5 | 0 | |  | Urine glucose (dispstix or benedicts test) | 1 | 0.5 | 0 | |  | Urine protein( disptix or acetic acid) | 1 | 0.5 | 0 | |  | HIV (rapid, ELISA or western Blott) | 1 | 0.5 | 0 | |  | AFB for TB | 1 | 0.5 | 0 | |  | Syphilis (VDRL or RPR) | 1 | 0.5 | 0 | |
| **PHARMACEUTICAL (Trace drugs)** |
| |  | | | | | --- | --- | --- | --- | |  | | **Present with at least one unit with valid date of expiration** | **No valid unit present** | |  | Amoxicillin suspension for children | 1 | 0 | |  | Amoxicillin tablets or capsules | 1 | 0 | |  | First-line antimalarial drugs(Coaterm) | 1 | 0 | |  | Co-trimoxazole for prophylaxis | 1 | 0 | |  | Oral or injectable contraceptives | 1 | 0 | |  | Any anti hypertensive drugs(Frusemide,propranolo) | 1 | 0 | |  | ARVS FOR PMTCT For example, AZT or NVP | 1 | 0 | |  | Country first-line ART for HIV | 1 | 0 | |  | Country first-line TB drug (Fixed dose or individuals) | 1 | 0 | |  | Insulin | 1 | 0 | |  | Paracetamol | 1 | 0 | |  | Oral rehydration salts (ORS) | 1 | 0 | |  | Vitamin A capsules (any dose) | 1 | 0 | |  | Folic acid (may be combined with iron) | 1 | 0 | |  | Iron tables (may be combined with folic acid) | 1 | 0 | |  | Salbutamol | 1 | 0 | |  | Food supplements for children | 1 | 0 | |  | Food supplement for adults | 1 | 0 | |  | Utero tonic(e.g.Oxtocin) | 1 | 0 | |  | DPT 3 vaccine | 1 | 0 | |  | Tetanus toxoid vaccine | 1 | 0 | |
| |  | **Infection Control** | | **No** | | **Yes** |  | | --- | --- | --- | --- | --- | --- | --- | |  | Chlorine-based disinfectant | 0 | | 1 | | | |  | Latex gloves (clean or sterile) | 0 | | 1 | | | |  | Sharps container | 0 | | 1 | | | |  | 5 ml plastic syringe in sterile packet | 0 | | 1 | | | |  | 19- or 21-gauge needle in sterile packet (may be  with syringe) | 0 | | 1 | | | |  | Hand-washing soap (bar or liquid) | 0 | | 1 | | |  | Disposal of hazardous waste materials | | --- | |
| |  |  | | **Yes** | | --- | --- | --- | --- | |  | Do you have an INCINERATOR No | | 0 | |  |  | Yes | 1 | |
| |  | **How do you dispose INFECTED WASTE** |  | | --- | --- | --- | |  | BURNING AND BURYING | 1 | |  | BURNING ONLY | 0.5 | |  | NOTHING DONE | 0 | |
|  |
| |  | **How do you dispose INFECTED SHARPS** | | | | --- | --- | --- | --- | |  | | BURNING AND BURYING | 1 | |  | | BURNING ONLY | 0.5 | |  | | NOTHING DONE | 0 |   0  No   1. **Any obvious medical waste in the surroundings**   1  Yes |

**Total score**
