# Supplementary material for: Application of Balanced Scorecard in the Evaluation of a Complex Health System Intervention: 12 Months Post Intervention Findings from the BHOMA Intervention: A Cluster Randomised Trial in Zambia
Source: PLoS One. 2014 Apr 21;9(4):e93977. doi: 10.1371/journal.pone.0093977 (PMC3994016; doi:10.1371/journal.pone.0093977)
Supplement: Tools S2 — Adult Clinical observation checklist. (DOC) [file pone.0093977.s002.doc]

| BACKGROUND INFORMATION | | | | | | | | | | | | | | | | | | | | | | | | | |  | |
| --- | --- | --- | --- | --- | --- | --- | --- | --- | --- | --- | --- | --- | --- | --- | --- | --- | --- | --- | --- | --- | --- | --- | --- | --- | --- | --- | --- |
| Instructions: This questionnaire is to be administered to health workers who are seeing the patients at the time of the Facility Study | | | | | | | | | | | | | | | | | | | | | | | | | | |  |
|  |  |  | | | | |  | | | | | | | | | | | |  | | | | | | | | |
|  | HF_ID | Health Facility ID | | | | | | | | | | |  | |  | | |  | | | | |  | | | | |
|  |  | | | | | | | | | | | | | | | | | | | | | | | | | | |
|  | HF_NAM | | **Health Facility Name** | | | | | |  | | | | | | | | | | | | | | | | | | |
|  |  | | | | | | | | | | | | | | | | | | | | | | | | | | |
|  | NAi | | **Name of Interviewer** | | | | |  | | | | | | | | | | | | | | | | | | | |
|  |  | | | | | | | | | | | | | | | | | | | | | | | | | | |
|  |  | | | | | **D** | | | | **D** | **M** | | | **M** | | **Y** | | | | **Y** | | **Y** | | | **Y** | | |
|  | HF_AO _01 | Date of Visit | |  | |  | | | |  |  | | |  | |  | | | |  | |  | | |  | | |
|  |  |  | |  | | | | | | | |  | | | | | | | | | | | | | | | |
|  | HF_AO _02 | Observation | |  | | | | | | | | | | | | |  | | | | Of | | |  | | | |
|  |  |  | |  |  | | | | | | | | | | | | | | | | | | | | | | |
|  | HF_AO _03 | Cadre | |  |  | | | | | | | | | | | | | | | | | | | | | | |
|  |  |  | |  | | | | | | | |  | | | | | | | | | | | | | | | |

|  | | **Male** | **Female** |
| --- | --- | --- | --- |
| HF_AO_04 | Sex | 1 | 2 |

| HF_AE_05 | **Age in years** |  |  |  |
| --- | --- | --- | --- | --- |

| HF_AO_06 | **Department/Section** | | Outpatient | | 1 |
| --- | --- | --- | --- | --- | --- |
|  |  | | ART Clinic | | 2 |
|  |  | | VCT | | 3 |
|  |  | | Antenatal (MCH) | | 4 |
|  |  | | TB Clinic | | 5 |
|  |  | | Other | | 6 |
|  | | Specify | |  | |

| HF_AO_07_1 | **Time began** |  | |  |  | | : |  |  |
| --- | --- | --- | --- | --- | --- | --- | --- | --- | --- |
|  | | | | | | | | | |
| HF_AO _07_2 | **Time ended** |  | |  |  | | : |  |  |
|  | | | | | | | | | |
| HF_AO _07_3 | **Total visit time** |  |  | | |  | |  |  |

|  |
| --- |

| HF_AO _08 | **Language of session** | Nyanga | | 1 |
| --- | --- | --- | --- | --- |
|  |  | Bemba | | 2 |
|  |  | English | | 3 |
|  |  | Other | | 4 |
|  | | Specify |  | |

| HF_AO _09 | **Main complaint (Check all that apply)** | | Cough | | 1 | |
| --- | --- | --- | --- | --- | --- | --- |
|  |  | | Fever | | 2 | |
|  |  | | Headache | | 3 |  |
|  |  | | Abdominal pain | | 4 |  |
|  |  | | ARV Treatment | | 5 |  |
|  |  | | Antenatal | | 6 |  |
|  |  | | Voluntary Testing and Counselling | | 7 |  |
|  |  | | Other | | 8 |  |
|  | | Specify | |  | | |

| HF_AO_10 | **Type of visit (Check that apply)** | | | | | |
| --- | --- | --- | --- | --- | --- | --- |
|  |  | Initial visit (new problem or new referred partner) | | | 1 | |
|  |  | Follow up visit (Previously diagnosed problem) | | | 2 | |
|  |  | Other | | | 3 | |
|  | | | Specify |  | |  |

| WELCOME AND RAPPORT WITH PATIENT/CLIENT | | | | | | | |  | |
| --- | --- | --- | --- | --- | --- | --- | --- | --- | --- |
|  | HF_AO_11 | | **Did the health worker?** | | | | | | |
|  |  | |  | | **No** | **Yes** | **N/A** | | |
|  |  | HF_AO_11_1 | | Receive patient in welcoming manner | 0 | 1 | 9 | |  |
|  |  | HF_AO_11_2 | | Introduce self to patient | 0 | 1 | 9 | |  |
|  |  | HF_AO_11_3 | | Offer patient a seat | 0 | 1 | 9 | |  |
|  |  | HF_AO_11_4 | | Explain presence of observer | 0 | 1 | 9 | |  |
|  |  | HF_AO_11_5 | | Obtain consent from the patient for the observer to be there | 0 | 1 | 9 | |  |

| HISTORY TAKING | | | | | | | |  | |
| --- | --- | --- | --- | --- | --- | --- | --- | --- | --- |
|  |  | | |  | | |  | | |
|  | HF_AO_12 | | **Did the health worker ask about?** | | **No** | **Yes** | **N/A** | | |
|  |  | HF_AO_12_1 | Ask about the presenting problem | | 0 | 1 | 9 | |  |
|  |  | HF_AO_12_2 | Ask about past medical history | | 0 | 1 | 9 | |  |
|  |  | HF_AO_12_3 | Ask about HIV status | | 0 | 1 | 9 | |  |
|  |  | HF_AO_12_4 | Identify danger signs (Respiratory, convulsions, severe pain, fever >39°C) | | 0 | 1 | 9 | |  |

|  |  | | **No** | **Yes** |
| --- | --- | --- | --- | --- |
| HF_AO_13 | | **Was physical examination done (If NO, go to** HF_AO_15_1) | 0 | 1 |

| HF_AO_14 | | **If YES, Did the health worker** | | | **No** | **Yes** | | **N/A** |
| --- | --- | --- | --- | --- | --- | --- | --- | --- |
|  | HF_AO_14_1 | | Explain rationale and procedure for physical examination | | 0 | 1 | | 9 |
|  | HF_AO_14_2 | | Ensure patient has privacy | | 0 | 1 | | 9 |
|  | HF_AO_14_3 | | Prepare the instruments before exam | | 0 | 1 | | 9 |
|  | HF_AO_14_4 | | Wash hands before exam | | 0 | 1 | | 9 |
|  | HF_AO_14_5 | | Perform a general examination | | 0 | 1 | | 9 |
|  | HF_AO_14_6 | | Perform obstetric examinations | | 0 | 1 | | 9 |
|  | HF_AO_14_7 | | Thank the patient after examination | | 0 | 1 | | 9 |
|  | HF_AO_14_8 | | Order appropriate investigation | | 0 | 1 | | 9 |
|  | | | |  | | |  | |

| DIAGNOSIS |
| --- |

| HF_AO_15 | | **Diagnosis (Health Worker’s diagnosis)** | | | | | |
| --- | --- | --- | --- | --- | --- | --- | --- |
|  | HF_AO_15_1 | | HIV | | **No** | **Yes** | **N/A** |
|  | HF_AO_15_2 | | Tuberculosis | | 0 | 1 | 9 |
|  | HF_AO_15_3 | | Malaria | | 0 | 1 | 9 |
|  | HF_AO_15_4 | | Pregnancy related | | 0 | 1 | 9 |
|  | HF_AO_15_5 | | Hypertension | | 0 | 1 | 9 |
|  | HF_AO_15_6 | | Diabetes | | 0 | 1 | 9 |
|  | HF_AO_15_7 | | Other | | 0 | 1 | 9 |
|  | HF_AO_15_8 | | **Specify** |  | | | |

| HF_AO_16 | | **Observer’s diagnosis** | | | | | | | |
| --- | --- | --- | --- | --- | --- | --- | --- | --- | --- |
|  | HF_AO_16_1 | | HIV | | **No** | **Yes** | **N/A** | | |
|  | HF_AO_16_2 | | Tuberculosis | | 0 | 1 | | | 9 |
|  | HF_AO_16_3 | | Malaria | | 0 | 1 | | | 9 |
|  | HF_AO_16_4 | | Pregnancy related | | 0 | 1 | | | 9 |
|  | HF_AO_16_5 | | Hypertension | | 0 | 1 | | | 9 |
|  | HF_AO_16_6 | | Diabetes | | 0 | 1 | | 9 | |
|  | HF_AO_16_7 | | Other | | 0 | 1 | | 9 | |
|  | HF_AO_16_8 | | Specify |  | | | | | |

| MANAGEMENT |
| --- |

| HF_AO_17 | |  | | **No** | **Yes** |
| --- | --- | --- | --- | --- | --- |
|  | HF_AO_17_1 | | Observer agrees with health worker | 0 | 1 |
|  | HF_AO_17_2 | | Observer diagrees with health worker | 0 | 1 |
|  | HF_AO_17_3 | | Observer not sure of management plan | 0 | 1 |
|  | HF_AO_17_4 | | Health worker not sure of management plan | 0 | 1 |

| HF_AO_18 | **Did observer prompt health worker to reconsider treatment?** | | **No** | **Yes** | **N/A** |
| --- | --- | --- | --- | --- | --- |
|  | |  | 0 | 1 | 9 |

| HF_AO_19 | **Did the health worker explain the treatment to the patient?** | | **No** | **Yes** | **N/A** |
| --- | --- | --- | --- | --- | --- |
|  | |  | 0 | 1 | 9 |

| HF_AO_20 | **Did the health worker give the next appointment for the patient?** | | **No** | **Yes** | **N/A** |
| --- | --- | --- | --- | --- | --- |
|  | |  | 0 | 1 | 9 |

| HF_AO_21 | **Did the health worker allow the patient/client to ask questions?** | | **No** | **Yes** | **N/A** | |
| --- | --- | --- | --- | --- | --- | --- |
|  | |  | 0 | 1 | 9 |  |

**THANK THE RESPONDENT FOR THEIR PARTICIPATION**

|  | Interviewer’s code | Date | | | | | | | | Signature |
| --- | --- | --- | --- | --- | --- | --- | --- | --- | --- | --- |
|  |  | d | d | m | m | y | y | y | y |  |
| Interviewer |  |  |  |  |  |  |  |  |  |  |
| Field Manager |  |  |  |  |  |  |  |  |  |  |
| 1^st^ data entry |  |  |  |  |  |  |  |  |  |  |
| 2^nd^ data entry |  |  |  |  |  |  |  |  |  |  |
