# Supplementary material for: Application of Balanced Scorecard in the Evaluation of a Complex Health System Intervention: 12 Months Post Intervention Findings from the BHOMA Intervention: A Cluster Randomised Trial in Zambia
Source: PLoS One. 2014 Apr 21;9(4):e93977. doi: 10.1371/journal.pone.0093977 (PMC3994016; doi:10.1371/journal.pone.0093977)
Supplement: Tools S3 — Children clinical observation checklist. (DOC) [file pone.0093977.s003.doc]

|  | | | |  | | | | |  | | | | | | | | | |  | | | | | |  |
| --- | --- | --- | --- | --- | --- | --- | --- | --- | --- | --- | --- | --- | --- | --- | --- | --- | --- | --- | --- | --- | --- | --- | --- | --- | --- |
| HF_ID | | | | Health Facility ID | | | | | | | | | |  | |  | | |  | | |  | | |  |
|  | | | | | | | | | | | | | | | | | | | | | | | |  | |
| HF_NAM | | **Health Facility Name** | | | | | | | |  | | | | | | | | | | | | | |  | |
|  | | | | | | | | | | | | | | | | | | | | | | | |  | |
| NAI | | | **Name of Interviewer** | | | | | | | |  | | | | | | | | | | | | |  | |
|  | | | | | | | | | | | | | | | | | | | | | | | |  | |
|  | | | | | | | **D** | **D** | | | | | **M** | | **M** | **Y** | | **Y** | | **Y** | | | **Y** |  | |
| HF_CO_01 | | | | Date of Visit | |  |  |  | | | | |  | |  |  | |  | |  | | |  |  | |
|  | | | |  | |  | | | | | |  | | | | | | | | | | | |  | |
| HF_CO_02_1 | | | | Starting time | |  | | | | | |  | | |  | | : |  | | |  | | | | |
|  | | | | | | | | | | | | | | | | | | | | | | | | | |
|  | HF_CO_02_2 | | | | Ending time |  | | | | | |  | | |  | | : |  | | |  | | | | |

HF_CO_02_3 **Total Time**

| READ CONSENT FORM TO HEALTH WORKER, READ CONSENT FORM TO THE CHOSEN CARETAKER/PATIENT BEFORE THEY ENTER THE CONSULTATION ROOM. OBSERVE FIVE CONSECUTIVE ELIGIBLE CLINICAL CASES, ELIGIBLE CASES ARE THOSE THAT ARE SICK CHILDREN, 1-59 MONTHS OF AGE. THEY SHOULD BE SEEN FOR ANY ONE (OR A COMBINATION) OF THE THREE FOLLOWING REASONS:  CHILDREN: MALARIA OR FEVER, ARI OR RAPID OR DIFFICULT BREATHING, DIARRHOEA  ADULTS: HIV/TB/HYPERTENSION  THERE IS A SEPARATE COLUMN FOR EACH OF THE FIVE CASES OBSERVED.  FOR EACH QUESTION, TICK YES, NO, OR NOT APPLICABLE |
| --- |

| HF_CO_03 | **Case Number** | |  |  |
| --- | --- | --- | --- | --- |
|  | | | | |
| HF_CO_04 | **Age of child (in completed months 1-59)** |  |  |  |

| HF_CO_05 | | **Reason for visit (Circle ALL that apply) Should only be for fever/malaria, cough/rapid or difficult breathing, and/or diarrhoea** | | |
| --- | --- | --- | --- | --- |
|  | HF_CO_05_1 | | Coughing/breathing problem | 1 |
|  | HF_CO_05_2 | | Fever/malaria | 2 |
|  | HF_CO_05_3 | | Diarrhoea | 3 |

| HF_CO_06 | | **Does the Health worker:** | | | | | | | | | **No** | | | |  | | | | | **Yes** | | | | |  |
| --- | --- | --- | --- | --- | --- | --- | --- | --- | --- | --- | --- | --- | --- | --- | --- | --- | --- | --- | --- | --- | --- | --- | --- | --- | --- |
|  | HF_CO_06_1 | | | | | Ask about the ability to feed or breastfeed | | | | | 0 | | | |  | | | | | 1 | | | | |  |
|  | HF_CO_06_2 | | | | | Ask whether the child vomits everything | | | | | 0 | | | |  | | | | | 1 | | | | |  |
|  | HF_CO_06_3 | | | | | Ask about the presence of convulsions | | | | | 0 | | | |  | | | | | 1 | | | | |  |
|  | | |  | |  | | | | | | | | | | | | | | | | | | | |  |
| HF_CO_07 | | | **Does the health worker:** | | | | | **No** | | | | | **Yes** | | |  | | | | | **NC** | | | |  |
|  | HF_CO_07_1 | | | | | | Check nutritional status on child health card | 0 | | | | | 1 | | |  | | | | | 2 | | | |  |
|  | HF_CO_07_2 | | | | | | Check vaccinations on child health card | 0 | | | | | 1 | | |  | | | | | 2 | | | |  |
|  | |  | |  | | | | | | | | | | | | | | | | | | | | |  |
| HF_CO_08 | | **Does the health worker classify the child as having :** | | | | | | | **No** | | | | | **Yes** | | | | **RDT** | | | | | **N/A** | |  |
|  | HF_CO_08_1 | | | | | | Fever of malaria | 0 | | | | | | 1 | | | | | 3 | | | | | 9 | |
|  | HF_CO_08_2 | | | | | | Pneumonia or fast/difficult breathing | 0 | | | | | | 1 | | | | |  | | | | | 9 | |
|  | HF_CO_08_3 | | | | | | Diarrhoea without blood | 0 | | | | | | 1 | | | | |  | | | | | 9 | |
|  | HF_CO_08_4 | | | | | | Diarrhoea with blood | 0 | | | | | | 1 | | | | |  | | | | | 9 | |
|  | |  | |  | | | | | | | | | | | | | | | | | | | | |  |
| HF_CO_09 | | **Does the health worker prescribe:** | | | | | | | | **No** | | | | **Yes** | | | |  | | | | | **N/A** | |  |
|  | HF_CO_09_1 | | | | | | First line anti malarial | | | 0 | | | | 1 | | | |  | | | | | 9 | |  |
|  | HF_CO_09_2 | | | | | | First line antibiotic for pneumonia | | | 0 | | | | 1 | | | |  | | | | | 9 | |  |
|  | HF_CO_09_3 | | | | | | ORS | | | 0 | | | | 1 | | | |  | | | | | 9 | |  |
|  | HF_CO_09_4 | | | | | | First line antibiotic for diarrhoea with blood | | | 0 | | | | 1 | | | |  | | | | | 9 | |  |
|  | HF_CO_09_5 | | | | | | Other antibiotic | | | 0 | | | | 1 | | | |  | | | | | 9 | |  |
|  | |  | |  | | | | | | | | | | | | | | | | | | | | |  |
|  | |  | |  | | | | | | | | | | | | | | | | | | | | |  |
| HF_CO_10 | | **Does the health worker explain how to administer:** | | | | | | | | **No** | | | | **Yes** | | | |  | | | | | **N/A** | |  |
|  | HF_CO_10_1 | | | | | | First line anti malarial? | | | 0 | | | | 1 | | | |  | | | | | 9 | |  |
|  | HF_CO_10_2 | | | | | | First line antibiotic for pneumonia? | | | 0 | | | | 1 | | | |  | | | | | 9 | |  |
|  | HF_CO_10_3 | | | | | | ORS? | | | 0 | | | | 1 | | | |  | | | | | 9 | |  |
|  | HF_CO_10_4 | | | | | | First line antibiotic for diarrhoea with blood | | | 0 | | | | 1 | | | |  | | | | | 9 | |  |
| HF_CO_11 | | **Does the health worker advise:** | | | | | | | | | | **No** | | | | | |  | | | | | **Yes** | |  |
|  | | About need to continue feeding during illness? | | | | | | | | | | 0 | | | | |  | | | | | 1 | | |  |

| **Supervisor Recode for Indicator #11 (HW performance - treatment):** Does classification (HF_CO_09) match the medication prescibed (HF_CO_10)? | | **CASE** | |  |  | |
| --- | --- | --- | --- | --- | --- | --- |
|  | |  |
| HF_CO_12_1 | Malaria or fever / first line antimalarial | Match | Not match | RDT | |  |
| HF_CO_12_2 | Pneumonia or difficult breathing / first line antibiotic for pneumonia | Match | Not match |  | |  |
| HF_CO_12_3 | Diarrhoea **without** blood / ORS but no antibiotic | Match | Not match |  | |  |
| HF_CO_12_4 | Diarrhoea **with** blood / first line antibiotic for dysentery | Match | Not match |  | |  |
| HF_CO_13 | **INDICATOR #11 (numerator = all match)** | All match | Not all match | RDT done | |  |

| HF_CO_14 | NOTE ANY QUALITATIVE OBSERVATIONS HERE: |  |
| --- | --- | --- |

**THANK THE RESPONDENT FOR THEIR PARTICIPATION**

|  | Interviewer’s code | Date | | | | | | | | Signature |
| --- | --- | --- | --- | --- | --- | --- | --- | --- | --- | --- |
|  |  | d | d | m | m | y | y | y | y |  |
| Interviewer |  |  |  |  |  |  |  |  |  |  |
| Field Manager |  |  |  |  |  |  |  |  |  |  |
| 1^st^ data entry |  |  |  |  |  |  |  |  |  |  |
| 2^nd^ data entry |  |  |  |  |  |  |  |  |  |  |
