# Supplementary material for: Application of Balanced Scorecard in the Evaluation of a Complex Health System Intervention: 12 Months Post Intervention Findings from the BHOMA Intervention: A Cluster Randomised Trial in Zambia
Source: PLoS One. 2014 Apr 21;9(4):e93977. doi: 10.1371/journal.pone.0093977 (PMC3994016; doi:10.1371/journal.pone.0093977)
Supplement: Tools S4 — Finance tool. (DOC) [file pone.0093977.s004.doc]

|  |  | | | | | | | | | | | |  | | | | | | | | | | |  | | | | | | |
| --- | --- | --- | --- | --- | --- | --- | --- | --- | --- | --- | --- | --- | --- | --- | --- | --- | --- | --- | --- | --- | --- | --- | --- | --- | --- | --- | --- | --- | --- | --- |
| HF_ID | Health Facility ID | | | | | | | | | | | | | | | |  | | | |  | | |  | | | |  | | |
|  | | | | | | | | | | | | | | | | | | | | | | | | | | | | | | |
| HF_NAM | Health Facility Name | | |  | | | |  | | | | | | | | | | | | | | | | | | | | | | |
|  | | | | | | | | | | | | | | | | | | | | | | | | | | | | | | |
| HF_DIS | **District** | | | | | | |  | | | | | | | | | | | | | | | | | | | | | | |
|  | | | | | | | | | | | | | | | | | | | | | | | | | | | | | | |
| NAI | **Name of Interviewer** | | | | | | |  | | | | | | | | | | | | | | | | | | | | | | |
|  | | | | | | | | | | | | | | | | | | | | | | | | | | | | | | |
|  | | | | | | | | | | **D** | | **D** | | | **M** | | | **M** | | | **Y** | | **Y** | | | **Y** | | | **Y** | |
| HF_FN_01 | Date of Visit | | | | |  | | | |  | |  | | |  | | |  | | |  | |  | | |  | | |  | |
|  | | | | | | | | | | | | | | | | | | | | | | | | | | | | | | |
|  | | | | | | | | | | | | | | | | | | | | | | | | | | | | | | |
| HF_FN_02 | GPS Coordinates | Latitude | S | | - | |  | |  | | ° | | |  | |  | | | ‚ |  | |  | | |  | | ‘ | | |  |
|  |  | Longitude | E | | 0 | |  | |  | | ° | | |  | |  | | | ‚ |  | |  | | |  | | ‘ | | |  |
|  | | | | | | | | | | | | | | | | | | | | | | | | | | | | | | |

| Instructions: Enter the amount of money in the boxes provided. If no records are available indicate “No record”. If not sure indicate “Not Sure”. If not applicable write “N/A” |
| --- |

| HF_FN_03 | **Do you have an action plan for the last 12 months?** | | |  |
| --- | --- | --- | --- | --- |
|  |  | No | 0 |  |
|  |  | Yes, copy seen | 1 | |
|  |  | Yes, no copy seen | 2 | |

| HF_FN_04 | **Do you have a person who is full time or parttime in charge of financing section at this health facility?** | | |  |
| --- | --- | --- | --- | --- |
|  |  | No | 0 |  |
|  |  | Yes | 1 | |

| HF_FN_04_1 | **If yes, has this person received any training in Finance Management in the last 12 months? (If NO or NOT SURE, go to HF_FN_05_1)** | | |
| --- | --- | --- | --- |
|  |  | No | 0 |
|  |  | Yes | 1 |
|  |  | Not sure | 9 |

| HF_FN_05 | **Indicate how much was received from the amount you budgeted in the last 12 months?** | | | | | | | | | | |
| --- | --- | --- | --- | --- | --- | --- | --- | --- | --- | --- | --- |
|  | | Budgeted |  |  |  |  |  | |  |  |  |
|  | | | | | | | | | | | |
|  | | Received |  |  |  |  |  | |  |  |  |
|  | | | | | | | | | | | |
| HF_FN_06 | **The last disbursement you received, how long did it take for you to receive the money from time of requesting?** | | | | | | |  | | | |

| HF_FN_07 | **Do you charge patients for any user fees?** | | |
| --- | --- | --- | --- |
|  |  | No | 0 |
|  |  | Yes | 1 |
|  |  | Not sure | 9 |

|  | | **No** | **Yes** |
| --- | --- | --- | --- |
| HF_FN_25 | **Did you receive any form of donation/payment in kind?** | 0 | 1 |

|  | | D_FN_26_1 | If yes to HF_FN_25, can you list down all the items received in the last 12 months and their quantities? | | | | | | | | | |
| --- | --- | --- | --- | --- | --- | --- | --- | --- | --- | --- | --- | --- |
|  | **Item & Qty** | | | **Donor** | **Amount (ZMK) where possible** | | | | | | | |
|  |  | | |  |  |  |  |  |  |  |  |  |
|  | | | | | | | | | | | | |
|  |  | | |  |  |  |  |  |  |  |  |  |
|  | | | | | | | | | | | | |
|  |  | | |  |  |  |  |  |  |  |  |  |

**THANK THE RESPONDENT FOR THEIR PARTICIPATION**

|  | Interviewer’s code | Date | | | | | | | | Signature |
| --- | --- | --- | --- | --- | --- | --- | --- | --- | --- | --- |
|  |  | d | d | m | m | y | y | y | y |  |
| Interviewer |  |  |  |  |  |  |  |  |  |  |
| Field Manager |  |  |  |  |  |  |  |  |  |  |
| 1^st^ data entry |  |  |  |  |  |  |  |  |  |  |
| 2^nd^ data entry |  |  |  |  |  |  |  |  |  |  |
