# Supplementary material for: Application of Balanced Scorecard in the Evaluation of a Complex Health System Intervention: 12 Months Post Intervention Findings from the BHOMA Intervention: A Cluster Randomised Trial in Zambia
Source: PLoS One. 2014 Apr 21;9(4):e93977. doi: 10.1371/journal.pone.0093977 (PMC3994016; doi:10.1371/journal.pone.0093977)
Supplement: Tools S5 — Governance tool. (DOC) [file pone.0093977.s005.doc]

| TO BE ADMINISTERED TO HEALTH FACILITY | | | | | | | | | | | | | | | | | |  |
| --- | --- | --- | --- | --- | --- | --- | --- | --- | --- | --- | --- | --- | --- | --- | --- | --- | --- | --- |
|  |  |  | | | | |  | | | | | | |  | | | | |
|  | HF_ID | Health Facility ID | | | | | | |  | |  | |  | | |  | | |
|  |  | | | | | | | | | | | | | | | | | |
|  | HF_NAM | **Health Facility Name** | |  | | | | | | | | | | | | | | |
|  |  | | | | | | | | | | | | | | | | | |
|  | NAI | **Name of Interviewer** | |  | | | | | | | | | | | | | | |
|  |  | | | | | | | | | | | | | | | | | |
|  |  | | | | **D** | **D** | | **M** | | **M** | **Y** | **Y** | | | **Y** | | **Y** | |
|  | Q01_DAT | Date of Visit |  | |  |  | |  | |  |  |  | | |  | |  | |
|  |  | | | | | | | | | | | | | | | | | |

| Statements of Good Health Governacne Practice  Indtructionhs: PLEASE FILL IN THIS SELF ASSESSMENT FORM. SAY WHETHER YOU AGREE OR DISAGREE WITH THE FOLLOWING STATEMENTS IN RELATION TO WHAT HAPPENS AT THIS HEALTH FACILITY | |
| --- | --- |
| **4=Agree 3=Some what agree 2=Some what disagree 1=Disagree** |  |

|  | **Vision** |  |  | | |  |  | | |
| --- | --- | --- | --- | --- | --- | --- | --- | --- | --- |
| Q02_1_PRO | The health facility has protocols for adult, child and maternal health services from the MoH | | | 4 | 3 | | 2 | 1 | |
| Q02_2_LOI | Local organisations and health service users have influence on what services are offered at the health facility | | | 4 | 3 | | 2 | 1 | |
| Q02_3_FMP | The facility managers ensure that Health workers follow protocols, standards and codes of conduct | | | 4 | 3 | | 2 | 1 | |
| Q02_4_RRE | The health facilities receive regular external quality check team to ensure that the protocols and standards are followed | | | 4 | 3 | | 2 | 1 | |
|  | | | | | | | | | |
| Q02_5_VIS | Vision score (out of 16) | | | | | |  | |  |

|  | **Intelligience and oversight** | | | | | | |
| --- | --- | --- | --- | --- | --- | --- | --- |
| Q03_1_CAD | The health facility collects and analyses local data | 4 | 3 | | 2 | 1 | |
| Q03_2_RRD | Health facility managers rely on research data from health facility to plan services | 4 | 3 | | 2 | 1 | |
| Q03_3_IIS | The health facility use evidence on program results, patient satisfaction, and other health-related information to improve the services they deliver | 4 | 3 | | 2 | 1 | |
|  | | | | | | | |
| Q03_4_INS | **Intelligience score (out of 12)** | | |  | | |  |

|  | **Regulation and management capacity (fair rules of the game)** | | | | | | |
| --- | --- | --- | --- | --- | --- | --- | --- |
| Q04_1_MCC | There is a mechanism for correcting those not complying with standards and code of conduct | 4 | 3 | | 2 | 1 | |
| Q04_2_IPD | Health services are organised and financed in ways that offer incentives to health workers and community health workers to improve performance in the delivery of health services | 4 | 3 | | 2 | 1 | |
| Q04_3_PPP | There are forums and procedures that give the public, technical experts, and local communities’ opportunities to provide inputs into the development of priorities, strategies, plans, and budgets | 4 | 3 | | 2 | 1 | |
| Q04_4_AUR | The allocation and utilization of resources are regularly tracked and information on results is available for review by the local communities and concerned stakeholders. | 4 | 3 | | 2 | 1 | |
| Q04_5_SER | Systems exist for reporting, investigating, and adjudicating misallocation or misuse of resources. | 4 | 3 | | 2 | 1 | |
| Q04_6_OFS | The health facility regularly organize forums to solicit input from the public and concerned stakeholders (vulnerable groups, groups with particular health issues, etc.) about priorities, services, and resources. | 4 | 3 | | 2 | 1 | |
| Q04_7_CSQ | The public or concerned stakeholders have regular opportunities to meet with managers of the health facility to raise issues about service efficiency or quality | 4 | 3 | | 2 | 1 | |
| Q04_8_PPB | The public and concerned stakeholders have the capacity to advocate and participate effectively with the health facility officials in the establishment of policies, plans, and budgets for health services. | 4 | 3 | | 2 | 1 | |
| Q04_9_QCG | Information about the quality and cost of health services is publicly available to help clients make choices as to where they want to go for health services | 4 | 3 | | 2 | 1 | |
| Q04_10_INE | There are procedures and systems that clients, providers, and concerned stakeholders can use to fight bias and inequity in accessing health service | 4 | 3 | | 2 | 1 | |
|  | | | | | | | |
| Q04_11_RES | **Regulation Score:(out of 40)** | | |  | | |  |
|  | | | | | | | |
| Q04_12_TGS | **Total Governance Score (out of 68)** | | |  | | |  |

**THANK THE RESPONDENT FOR THEIR PARTICIPATION**

|  | Interviewer’s code | Date | | | | | | | | Signature |
| --- | --- | --- | --- | --- | --- | --- | --- | --- | --- | --- |
|  |  | d | d | m | m | y | y | y | y |  |
| Interviewer |  |  |  |  |  |  |  |  |  |  |
| Field Manager |  |  |  |  |  |  |  |  |  |  |
| 1^st^ data entry |  |  |  |  |  |  |  |  |  |  |
| 2^nd^ data entry |  |  |  |  |  |  |  |  |  |  |
