# Supplementary material for: Application of Balanced Scorecard in the Evaluation of a Complex Health System Intervention: 12 Months Post Intervention Findings from the BHOMA Intervention: A Cluster Randomised Trial in Zambia
Source: PLoS One. 2014 Apr 21;9(4):e93977. doi: 10.1371/journal.pone.0093977 (PMC3994016; doi:10.1371/journal.pone.0093977)
Supplement: Tools S6 — Health worker motivation tool. (DOC) [file pone.0093977.s006.doc]

| BACKGROUND INFORMATION | | | | | | | | | | | | | | | | | | | | |  |
| --- | --- | --- | --- | --- | --- | --- | --- | --- | --- | --- | --- | --- | --- | --- | --- | --- | --- | --- | --- | --- | --- |
|  | |  | | | | | |  | | | | | | | |  | | | | | |
| HF_ID | | Health Facility Code | | | | | | | | |  | | |  | | |  | |  | | |
|  | | | | | | | | | | | | | | | | | | | | | |
| HF_NAM | **Health Facility Name** | | | | |  | | | | | | | | | | | | | | | |
|  | | | | | | | | | | | | | | | | | | | | | |
| NAI | **Name of Interviewer** | | | |  | | | | | | | | | | | | | | | | |
|  | | | | | | | | | | | | | | | | | | | | | |
|  | | | | **D** | | | **D** | | | **M** | | **M** | **Y** | | **Y** | | | **Y** | | **Y** | |
| HF_HW_01 | | Date of Visit |  |  | | |  | | |  | |  |  | |  | | |  | |  | |
|  | |  |  | | | | | |  | | | | | | | | | | | | |

| **INSTRUCTIONS: Administer this questionnaire to Health Workers found at the Health Facility** | | | | | | |
| --- | --- | --- | --- | --- | --- | --- |
|  | |  | | |  | |
| **Demographic Characteristics** | | | | | | |
|  | | | | | | |
| HF_HW_02 | **Age in years** | |  |  | |  |
|  | | | | | | |
|  | | **Male** | | | **female** | |
| HF_HW_03 | Sex | 1 | | | 2 | |

|  | |  | | |  |  |
| --- | --- | --- | --- | --- | --- | --- |
| HF_HW_04 | **Cadre** | | Nurse | | 1 |  |
|  |  | | Clinical Officer | | 2 |  |
|  |  | | EHT | | 3 | |
|  |  | | Doctor | | 4 | |
|  |  | | CDE | | 5 | |
|  |  | | Other | | 6 | |
|  | | | Specify |  | | |

|  | |  | |  |  |
| --- | --- | --- | --- | --- | --- |
| HF_HW_05 | **Time in the post** | | Upto 3 months ago | 1 |  |
|  |  | | 4-6 months ago | 2 |  |
|  |  | | 7-12 months ago | 3 | |
|  |  | | More than 12 months ago | 4 | |

| HF_HW_06 | | During the last 12 months, have you received any training **IF NO SKIP TO HF_HW_08** | | |
| --- | --- | --- | --- | --- |
|  |  | | **No** | **Yes** |
|  |  | | 0 | 1 |

| **Questions on motivation:**  **Do you strongly agree, agree, some what agree, disagree or strongly disagree with each of the following statements** | | | | | | | |
| --- | --- | --- | --- | --- | --- | --- | --- |
| HF_HW_10 | **General Motivation** | **Strongly agree** | **Agree** | **Some what agree** | **Disagree** | **Strongly disagree** | |
| HF_HW_10_1 | These days, I feel motivated to work as hard as I can | 5 | 4 | 3 | 2 | | 1 |
| HF_HW_10_2 | I only do this job so that I get paid at the end of the month | 5 | 4 | 3 | 2 | | 1 |
| HF_HW_10_3 | I do this job as it provides long term security for me | 5 | 4 | 3 | 2 | | 1 |

| HF_HW_11 | **Burnout** | | | | | |
| --- | --- | --- | --- | --- | --- | --- |
| HF_HW_11_1 | I feel emotionally drained at the end of the every day | 5 | 4 | 3 | 2 | 1 |
| HF_HW_11_2 | Sometimes when I get up in the morning, I dread having to face another day at work | 5 | 4 | 3 | 2 | 1 |

| HF_HW_12 | **Job satisfaction** | | | | | |
| --- | --- | --- | --- | --- | --- | --- |
| HF_HW_12_1 | Overall, I am very satisfied with my job | 5 | 4 | 3 | 2 | 1 |
| HF_HW_12_2 | I am not satified with my colleagues in my work | 5 | 4 | 3 | 2 | 1 |
| HF_HW_12_3 | I am satisfied with my supervisor | 5 | 4 | 3 | 2 | 1 |

| HF_HW_13 | **Intrinsic job satisfaction** | | | | | |
| --- | --- | --- | --- | --- | --- | --- |
| HF_HW_13_1 | I am satisfied with the opportunity to use my abilities in this job | 5 | 4 | 3 | 2 | 1 |
| HF_HW_13_2 | I am satisfied that I accomplish something worthwhile in this job | 5 | 4 | 3 | 2 | 1 |
| HF_HW_13_3 | I do not think that my work in this health facility is valuable these days | 5 | 4 | 3 | 2 | 1 |

| HF_HW_14 | **Organisational commitment** | | | | | |
| --- | --- | --- | --- | --- | --- | --- |
| HF_HW_14_1 | I am proud to be working for this health facility | 5 | 4 | 3 | 2 | 1 |
| HF_HW_14_2 | I find that my values and this health facility are very similar | 5 | 4 | 3 | 2 | 1 |
| HF_HW_14_3 | I am glad that I work for this facility rather than other facilities in the country | 5 | 4 | 3 | 2 | 1 |
| HF_HW_14_4 | I feel very little commitment to this health facility | 5 | 4 | 3 | 2 | 1 |
| HF_HW_14_5 | This health facility really inspires me to do my very best on the job. | 5 | 4 | 3 | 2 | 1 |

|  | **Conscientiousness** | | | | | | |
| --- | --- | --- | --- | --- | --- | --- | --- |
| HF_HW_15_1 | | I cannot be relied on by my colleagues at work | 5 | 4 | 3 | 2 | 1 |
| HF_HW_15_2 | | I always complete my tasks efficiently and correctly | 5 | 4 | 3 | 2 | 1 |
| HF_HW_15_3 | | I am a hard worker | 5 | 4 | 3 | 2 | 1 |
| HF_HW_15_4 | | Do things that need doing without being asked or told | 5 | 4 | 3 | 2 | 1 |

|  | **Timelines and attendance** | | | | | |
| --- | --- | --- | --- | --- | --- | --- |
| HF_HW_16_1 | I am punctual about coming to work | 5 | 4 | 3 | 2 | 1 |
| HF_HW_16_2 | I am often absent form work | 5 | 4 | 3 | 2 | 1 |
| HF_HW_16_3 | It is not a problem if I sometimes come late for work | 5 | 4 | 3 | 2 | 1 |

**THANK THE RESPONDENT FOR THEIR PARTICIPATION**

|  | Interviewer’s code | Date | | | | | | | | Signature |
| --- | --- | --- | --- | --- | --- | --- | --- | --- | --- | --- |
|  |  | d | d | m | m | y | y | y | y |  |
| Interviewer |  |  |  |  |  |  |  |  |  |  |
| Field Manager |  |  |  |  |  |  |  |  |  |  |
| 1^st^ data entry |  |  |  |  |  |  |  |  |  |  |
| 2^nd^ data entry |  |  |  |  |  |  |  |  |  |  |
