# Supplementary material for: Application of Balanced Scorecard in the Evaluation of a Complex Health System Intervention: 12 Months Post Intervention Findings from the BHOMA Intervention: A Cluster Randomised Trial in Zambia
Source: PLoS One. 2014 Apr 21;9(4):e93977. doi: 10.1371/journal.pone.0093977 (PMC3994016; doi:10.1371/journal.pone.0093977)
Supplement: Tools S7 — Service satisfaction for adults and children. (DOC) [file pone.0093977.s007.doc]

| **Service satisfaction indicators for adults and Children** | | | | | | |
| --- | --- | --- | --- | --- | --- | --- |
|  | | | | | | |
|  | For each of the following questions, I would like you to tell me if you thought the service was very good, good, fair, or poor. | Very poor | Poor | Fair | Good | Very good |
| **1** | The time you had to wait to be seen. Was this very good, good, fair, or Poor or very poor? |  |  |  |  |  |
| **2** | The explanation you received of your illness/child's illness. Was this very good, good, fair, poor or very poor ? |  |  |  |  |  |
| **3** | The treatment you received for your illness/child's illness. Was this very good, good, fair, poor or very poor? |  |  |  |  |  |
|  | *NB: Total scores were converted to percentage to generate scores out of 100.Adapted from WHO health facility questionnaire SPA* | | | | | |
