# Supplementary material for: Application of Balanced Scorecard in the Evaluation of a Complex Health System Intervention: 12 Months Post Intervention Findings from the BHOMA Intervention: A Cluster Randomised Trial in Zambia
Source: PLoS One. 2014 Apr 21;9(4):e93977. doi: 10.1371/journal.pone.0093977 (PMC3994016; doi:10.1371/journal.pone.0093977)
Supplement: Protocol S1 — Published protocol. (PDF) [file pone.0093977.s009.pdf]

Stringer et al. BMC Health Services Research 2013, 13(Suppl 2):S7
http://www.biomedcentral.com/1472-6963/13/S2/S7

BMC Health Services Research

STUDY PROTOCOL — Open Access

Protocol-driven primary care and community linkages to improve population health in rural Zambia: the Better Health Outcomes through Mentoring and Assessment (BHOMA) project

Jeffrey SA Stringer[1*], Angela Chisembele-Taylor[2], Carla J Chibwesha[2], Harmony F Chi[3], Helen Ayles[4], Handson Manda[2], Wendy Mazimba[2], Linnaea Schuttner[2], Ntazana Sindano[2], Frank B Williams[5], Namwinga Chintu[2], Roma Chilengi[2]

Abstract

Introduction: Zambia's under-resourced public health system will not be able to deliver on its health-related Millennium Development Goals without a substantial acceleration in mortality reduction. Reducing mortality will depend not only upon increasing access to health care but also upon improving the quality of care that is delivered. Our project proposes to improve the quality of clinical care and to improve utilization of that care, through a targeted quality improvement (QI) intervention delivered at the facility and community level.

Description of implementation: The project is being carried out 42 primary health care facilities that serve a largely rural population of more than 450,000 in Zambia's Lusaka Province. We have deployed six QI teams to implement consensus clinical protocols, forms, and systems at each site. The QI teams define new clinical quality expectations and provide tools needed to deliver on those expectations. They also monitor the care that is provided and mentor facility staff to improve care quality. We also engage community health workers to actively refer and follow up patients.

Evaluation design: Project implementation occurs over a period of four years in a stepped expansion to six randomly selected new facilities every three months. Three annual household surveys will determine population estimates of age-standardized mortality and under-5 mortality in each community before, during, and after implementation. Surveys will also provide measures of childhood vaccine coverage, pregnancy care utilization, and general adult health. Health facility surveys will assess coverage of primary health interventions and measures of health system effectiveness.

Discussion: The patient-provider interaction is an important interface where the community and the health system meet. Our project aims to reduce population mortality by substantially improving this interaction. Our success will hinge upon the ability of mentoring and continuous QI to improve clinical service delivery. It will also be critical that once the quality of services improves, increasing proportions of the population will recognize their value and begin to utilize them.

* Correspondence: Jeffrey_stringer@med.unc.edu
[1]Department of Obstetrics and Gynecology, University of North Carolina School of Medicine, Chapel Hill, NC, USA
Full list of author information is available at the end of the article

© 2013 Stringer et al; licensee BioMed Central Ltd. This is an Open Access article distributed under the terms of the Creative Commons Attribution License (http://creativecommons.org/licenses/by/2.0), which permits unrestricted use, distribution, and reproduction in any medium, provided the original work is properly cited.

Introduction

Despite encouraging recent economic growth and political stability, the Republic of Zambia remains a poor nation, with substantial public health challenges [1]. A high burden of pregnancy and perinatal complications, childhood diseases, cardiovascular and other non-communicable diseases, accidents, and infectious diseases, such as AIDS, tuberculosis, and malaria contribute to a life expectancy for the average Zambian of just 48 years [2,3]. The organized response to this disease burden is overwhelmingly shouldered by government (private health care accounts for less than 5% of the total modern health care delivered in Zambia), and is delivered through a system that is chronically under-resourced and under-staffed [4-6]. For the past decade, more than half of the nation's total health expenditure [7] has come from external donor resources [8]. And with a health worker to population ratio well below the critical threshold of 2.3 per 1,000 people [9], the system faces a critical human resource challenge [9-11].

Although some health indicators have improved over the past few years [12,13], overall progress must accelerate substantially if Zambia is to meet its targets for the health related Millennium Development Goals (MDGs) [14]. The under-5 mortality rate has improved from 183 per 1,000 live births in 1990 to 111 per 1,000 live births in 2010 [13]. This reflects an encouraging average annual rate of reduction of 3.5% in the past decade, compared to only 1.5% in the preceding 10 years [13]. However, the rate of reduction remains far from what is needed to reach the MDG 4 (reduce child mortality) target of 63.6 per 1,000 live births. Similarly, the regional maternal mortality ratio has improved slightly from 850 per 100,000 live births in 1990 to 500 per 100,000 live births in 2010, but reflects insufficient progress towards the MDG 5 (improve maternal health) target of 162 per 100,000 live births by 2015 [14].

Access to basic public health services remains a significant barrier to achieving MDGs 4 and 5. According to Zambia's last available (2007) Demographic and Health Survey (DHS), less than two-thirds of children with signs of acute respiratory infection, fever, or diarrhea receive care from a health provider [15]. Fewer than half of women deliver with assistance from a skilled provider, and fewer than half receive any postnatal care. Nearly three-fourths of all women report serious problems accessing health care (82% of rural women and 62% of urban women). Further barriers to access include the belief that the facility will have no drugs available, personal financial resources needed to pay for treatment or medication co-payments, and lack of transport [15].

When Zambians do access primary health clinics, they may not receive the services that national guidelines indicate they should. Although the most recent DHS reports that 94% of women have more than one antenatal visit, only 36% reported receiving deworming prophylaxis, 23% a urine test, and 59% any blood test [15].

Although the focus of discussions on health tends to be about women and children, the general adult population health situation in Zambia reflects an unacceptably high mortality and disease burden. The age-adjusted adult mortality rate for the age range 15-49 years was 12.5 deaths per 1,000 years of exposure; this is during years 0 through six before the 2007 DHS. The rate is higher among women (13.2 deaths per 1,000 years of exposure) than men (11.9 deaths per 1,000 years of exposure) [15]. There is a dearth of information on the specific contributors of adult ill-health outside HIV/AIDS. Anecdotally, we are aware that tuberculosis, sexually transmitted disease, and non-communicable diseases (e.g., diabetes, hypertension, and malignancies) are important contributors.

In our clinical experience, only a minority of pregnant women in the capital city of Lusaka receives appropriate diagnostic testing and treatment for the high-risk maternal conditions of syphilis, anemia, malaria, and gestational hypertension. It stands to reason that patients who do not expect health centers to have health care providers, drugs, or tests may not make the often extraordinary efforts required to access care. When a patient arrives at a clinic and finds health care providers who feel ill-equipped to properly deliver essential health services, the experience contributes to a loss of trust in the health care system (Figure 1). This loss of trust may mean that patients delay seeking care, presenting with more acute, complex needs, which translates in turn to poorer clinical outcomes. These poor outcomes can, in turn, further erode trust in the system.

The Zambia PHIT Partnership project, known locally as Better Health Outcomes through Mentoring and Assessment (BHOMA) is a five-year project that aims to improve the quality of clinical service delivery and restore community confidence in the health system. BHOMA functions as a partnership led by the University of North Carolina-Chapel Hill, the Center for Infectious Disease Research in Zambia, and the ZAMBART Project. Our work focuses deliberately at the interface of the health care system and the community — where an individual patient meets an individual provider. The organizing principle of the BHOMA project is that the patient-provider interaction is the central business of health care, and any effort to improve health systems should be oriented toward ensuring that this interaction succeeds. Put simply, if the patient-provider interaction goes well, the patient is likely to have a good outcome. If it does not, that likelihood is diminished.

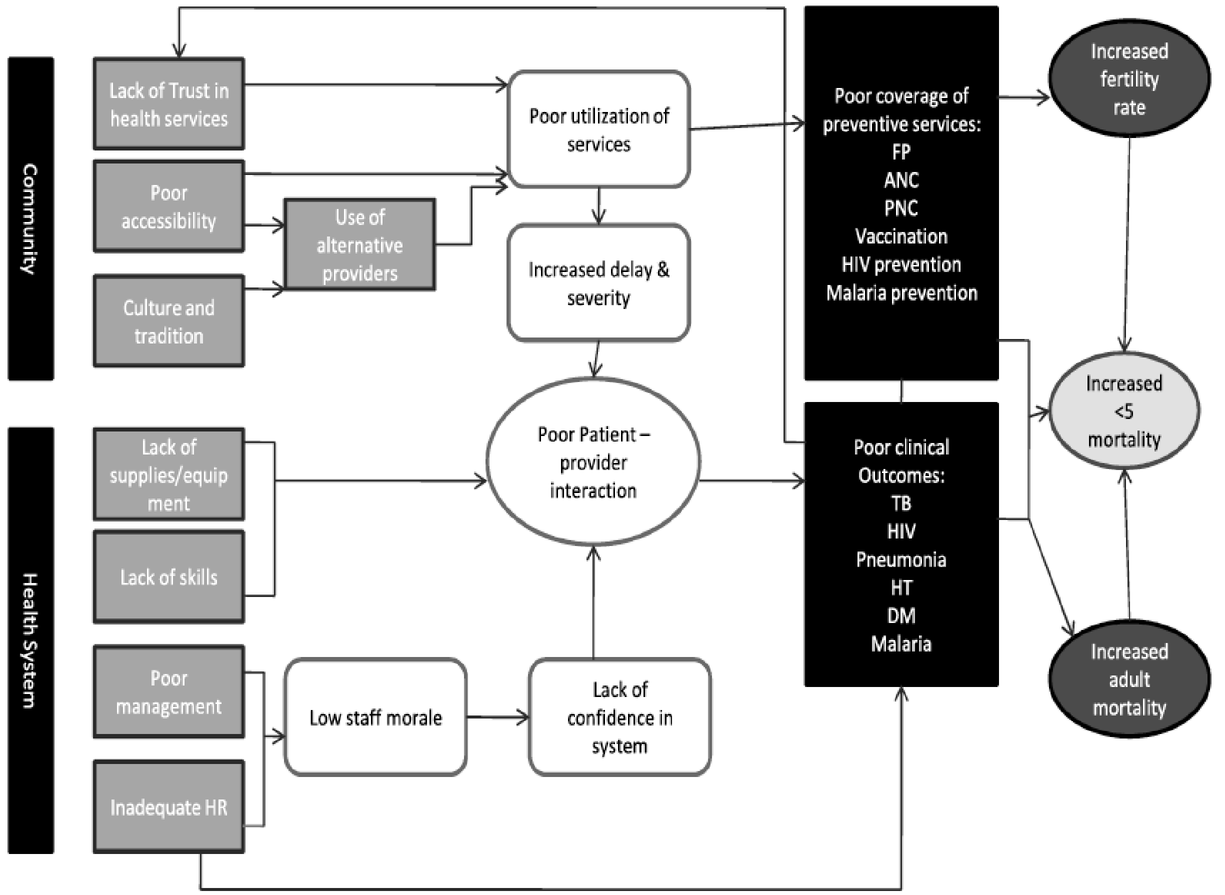

Figure 1 Conceptual framework for the BHOMA project. This figure demonstrates the conceptual framework of our intervention. The patient-provider interaction represents a critical interface at which the community meets the health system. If the interaction goes well, the patient is much more likely to have a good outcome. In addition to measuring the overall population mortality outcomes, our project has implemented data collect to quantify each of the intervening steps in the conceptual framework.

Our approach to improving the provider-patient interaction is to assess the clinical care provided at each visit and mentor clinicians to improve the care they routinely provide. Specifically, BHOMA's core objectives are to: 1) create a set of clear expectations for primary care through protocols and forms that guide providers at each visit; 2) ensure providers have the tools they need (equipment, supplies, diagnostics, and drugs) to deliver on what is asked of them; 3) monitor the care that is provided through an on-site electronic record that comprehensively and constantly measures clinical care quality; 4) improve performance of key indicators of clinical care quality by providing ongoing, on-site mentoring to develop better clinical skills and practices; and 5) increase community engagement with the health system through active patient referral and follow-up.

The design of the BHOMA intervention drew substantially from the systems and processes that were created to support Zambia's rapid and successful scale-up of HIV care and treatment, largely in primary health care settings. We postulate that a similar investment in clinical mentoring, data collection, and monitoring applied to primary care services could reverse the patterns of the past 20 years and produce measurable improvements in MDG and general health indicators for health.

Description of the implementation

We describe the project implementation below, according to its five core objectives. BHOMA has six dedicated quality improvement (QI) teams that lead field implementation activities. Each QI team includes a clinical officer and a nurse or midwife who have achieved "train the trainer" status in integrated management of adult illnesses (IMAI) [16-20], integrated management of childhood illnesses (IMCI)[2], and emergency obstetrical and newborn care (EmONC) [21]. The teams also include a pharmacist and data entry technician. Each QI team will work with one new health facility in a quarter and,

ultimately, support seven facilities over the course of the project. This allows teams to dedicate the necessary time to on-site implementation and mentoring and enables teams to establish long-term, supportive relationships with health facility staff. The teams work in the three, predominately rural districts of Lusaka Province (Kafue, Chongwe, and Luangwa) (Figure 2) that are home to more than 450,000 Zambians [22].

The first core objective is to create a set of clear expectations for primary care through protocols and forms that guide clinicians at each visit. The BHOMA team and its advisors, including key decision-makers in the Ministry of Health (MoH), reviewed extant national and international clinical care guidelines and created simple, step-by-step protocols for the diagnosis and management of common presentations encountered in rural primary care settings. In order to guide facility staff during patient visits and to ensure consistent documentation of all clinical encounters, we created a system of seven simple clinical forms. These include a patient registration form; a separate form for routine adult, pediatric, antenatal, neonatal, and delivery care; and a form to manage antenatal and delivery complications.

We introduce the protocols and forms at health facilities through intensive on-site training and system implementation that includes a review and revision of clinic processes and flows. During this "implementation visit," the QI team spends one month training staff in a variety of quality-related areas (Table 1). During the first two weeks of the implementation visit, the training focuses on diagnosis and management of common presentations and on introducing protocols for clinical management. (In most cases the care outlined in the protocols is not new, but it requires review in the context of the new forms and quality indicators that the QI team is introducing). All members of the facility are engaged at this stage of the training, including medical and nursing staff, clinic support workers, and community health workers. The content of the third and fourth week of training are detailed in the sections that follow.

The second objective is to ensure clinicians are equipped with a set of essential diagnostic and management tools that have been identified as necessary to provide quality care. BHOMA provides targeted logistic and funding support for essential supplies and equipment (e.g., sphygmomanometers, stethoscopes, baby scales).

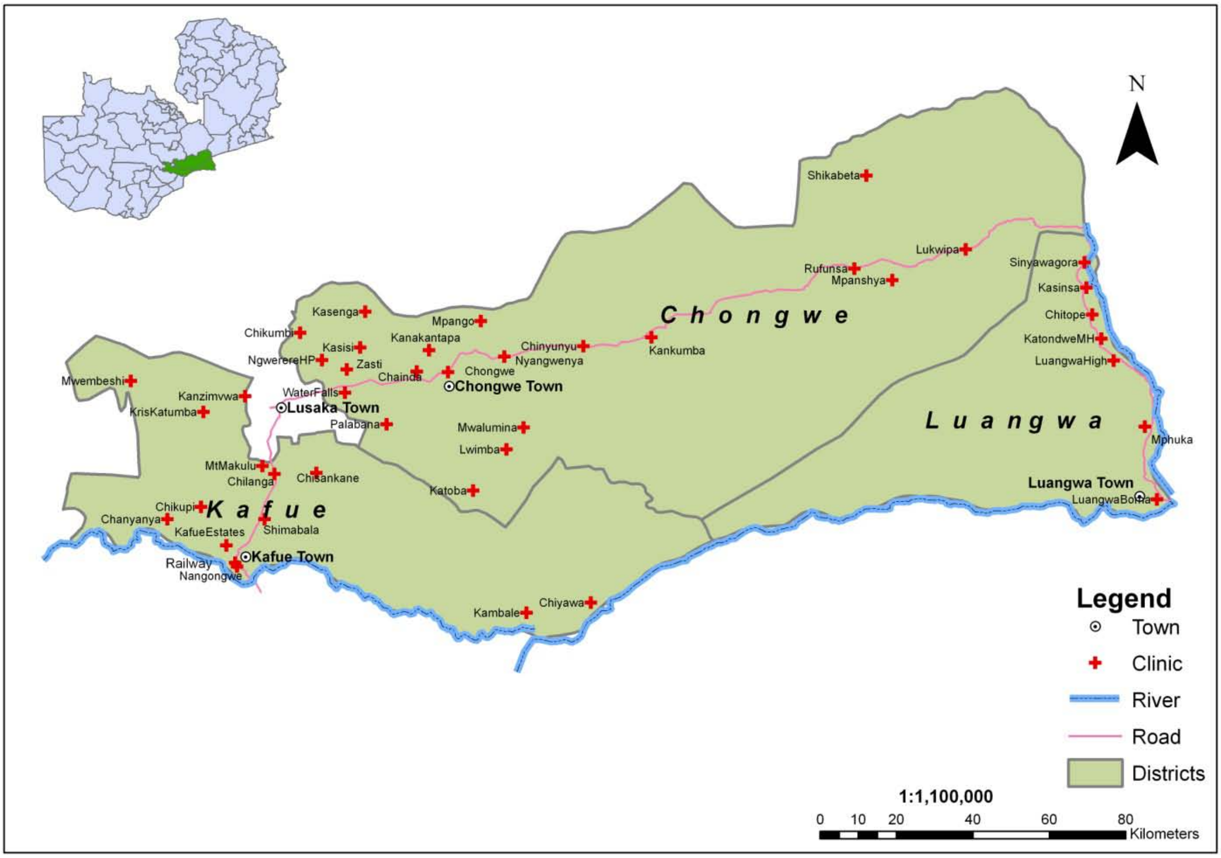

Figure 2 Map of BHOMA intervention districts with participating facilities indicated.

Table 1 Clinical Training Schedule

Week	Training Activities	Trainees
1 & 2	• Diagnosis and management of common presentations<br>• Clinical protocols	• Clinical staff<br>• Clinic support workers<br>• Community health workers
3	• Patient registration and triage<br>• Clinical forms<br>• Data entry<br>• Medical record keeping	• Clinical staff<br>• Clinic support workers<br>• Community health workers
4	• Patient registration and triage<br>• Clinical forms<br>• Data entry<br>• Medical record keeping<br>• Antenatal care, postnatal care, and danger signs during pregnancy	• Clinical staff<br>• Clinic support workers<br>• Community health workers<br>• Traditional birth attendants

After the QI team conducts pre-implementation facility assessments, the project procures and distributes essential missing equipment and supplies. On an ongoing basis, a BHOMA pharmacy technician based at each district health office (DHO) works to strengthen the district's ordering and supply system. This includes assistance with drug forecasting and ordering from central medical stores, fulfillment of requests for drug kits from outlying facilities, and comparing commodities usage reports across the district to identify potential weaknesses at the facility level.

The third objective is to monitor the care that is provided through an on-site electronic record that comprehensively and constantly measures clinical care quality and patient outcomes. During the third and fourth week of facility implementation, we establish an organized medical records system in which patients are assigned unique ID numbers where regular, organized, legible charts are kept at the facility for each patient. A new cadre of lay workers, known as "clinic support workers," supports the medical record system. The project employs two to three clinic support workers per facility, and they are responsible for a variety of tasks, including obtaining vital signs, checking in patients, collecting basic background information, and organizing and maintaining the clinic's medical record system. Clinic support workers ensure the relevant clinical forms are completed and filed in the patient's medical chart at the end of the clinical visit. They enter data from the clinical forms on a simple touch screen computer (model 615, J2 Retail Systems Inc., Irvine, CA, USA), and then file charts according to identification number to facilitate retrieval during subsequent clinic visits.

Authorized users at each facility can immediately see how well the facility is providing care according to a series of key performance indicators. Building substantially on experience implementing performance reports in HIV care and treatment services [23], we developed core performance indicators for adult, pediatric, and antenatal care visits. These performance metrics include simple indicators, such as the proportion of children whose full vital signs are recorded, the proportion of febrile children for whom a malaria rapid test result is recorded in the medical record, or the proportion of hypertensive pregnant women for whom the results of a urine protein dipstick is recorded. The metrics are designed to quantify the degree of adherence to clinical care protocols and serve a key role in clinical quality improvement. When the QI teams make their initial visit to a facility, all providers are trained in the creation and interpretation of performance reports. On subsequent visits, the reports are used as a starting point for clinical quality improvement. If, for instance, the performance reports indicate a high proportion of malaria diagnoses that have not received an appropriate antimalarial, then the QI team would work with the clinic staff to determine underlying causes (e.g., kit stock out and/or inappropriate management). In the case of the latter, malaria diagnosis and management would figure into the mentoring done at the next visit.

The fourth objective is to improve performance of key indicators of clinical care quality by providing ongoing, on-site mentoring to develop better clinical skills and practices. Intensive on-site mentorship provides continuing education to facility staff, strengthens clinical skills, and improves adherence to clinical guidelines. After the initial four-week implementation period, QI teams return to each new facility monthly for three months. After this, they return once a quarter to each facility. During these visits, QI teams conduct structured reviews of medical records, focusing on the accuracy of diagnosis and management. They review performance metrics with the facility's clinical officers, nurses, midwives, and other staff and work with them to develop specific goals and plans for improvement. This ongoing mentoring is coordinated by the DHOs, which plan and lead all clinical improvement activities. Our mentorship model builds on existing performance assessment and technical support structures, rather than creating a parallel support system for the project. Through dedicated staffing, the project also supports the implementation of existing district management tools, including the health management information system

(HMIS) and the District Integrated Logistics and Supplies Assessment Tool (DILSAT).

The fifth objective is to increase community engagement with the health system through active patient referral and follow-up by community health workers (CHWs) as well as traditional birth attendants (TBAs). BHOMA has recruited more than 200 CHWs, who participate in the four weeks of implementation training at each facility and receive mobile phones to assist with referral (Table 1). Each CHW receives training on recognition of danger signs and how to refer individuals and register the referral on their mobile phone. In keeping with Zambian national policy, CHWs are also authorized to dispense oral rehydration salts for management of diarrhea, folic acid and ferrous sulfate for management of anemia, and antimalarials.

All CHW phones are linked to the facility record system through mobile phone software known as CommCare®. When patients are registered at a facility, they are assigned to a CHW based upon where they live. If a patient does not return for a scheduled follow-up, the facility record system sends an alert to the CHW. Once at the patient's home, CommCare® guides the CHW through a series of questions to which they key in responses. This information, including patient outcomes, is transferred back to the clinic servers via mobile phone at the end of each household visit. In addition, CHWs visit all households in their assigned zones each quarter to conduct census surveys. They interview household heads, obtaining information about the number of individuals in the household, as well as pregnancies, illnesses, and deaths. Besides being documented in the central database, this information is also aggregated and used by neighborhood health committees (NHCs) to discuss local community health challenges and to address these challenges through local solutions.

To the extent reasonable, the project engages with already existing and MoH trained TBAs. The project only engages with untrained TBAs if there is a shortage of trained TBAs available in the particular NHC or zone. The identification and "recruitment" of TBA's on the BHOMA program is done in close collaboration with the Health Center in-charges. The TBA training consists of a one-week training that includes anti-partum, intrapartum, and postpartum care.

The rationale for the engaging TBAs is also to provide support to the shortage of qualified health workers in maternal child health (MCH) by task shifting. The TBAs are trained to assist the qualified health workers by carrying out duties in MCH, which includes vitals, urinalysis, and transcribing under supervision of qualified health workers. In the communities, their duties include early referral of pregnant mothers for antenatal care (ANC) visits; referral of pregnant mothers to institutional delivery; and a visit to monitor postnatal mothers during the first week and refer appropriately.

Evaluation design

The overall impact of the BHOMA intervention will be evaluated by means of a stepped wedge, cluster-randomized trial [24], where the intervention will be progressively introduced – in random order – to facilities across the three districts until all are providing the intervention. This progressive implementation will occur in seven "steps" of six facilities each, with each step separated by approximately three months. Since our implementation teams are district-based, and since the districts vary considerably in population and number of facilities eligible for randomization, we employed a stratification scheme to ensure that the same number of facilities in each district are implemented in each step. The Chongwe District will implement three facilities per step, the Kafue District two facilities per step, and the Luangwa District one facility per step. A statistician not otherwise involved in the study has randomly determined the order of implementation.

The co-primary outcomes of age-adjusted overall mortality and under-5 mortality are being assessed through a series of three household surveys conducted 12 months apart. The surveys allow population estimates of mortality in each community before, during, and after the implementation (Table 2).

Sampling

The BHOMA intervention is being implemented in 42 facilities and their surrounding communities. Each participating community is sampled in each of the three survey rounds. It is critical to our evaluation effort that we are able to definitively attribute a particular household's health outcomes to a particular facility. Any "contamination" that occurs as a result of our attributing outcomes from an adjacent control facility to an intervention facility would bias our study toward the null hypothesis. We therefore elected to use a "fried egg" design [25] to define our sampling frame, where the white of the egg represents a given facility's catchment area and the yolk represents the evaluation area (typically a 3.8 kilometer radius around the facility) where sampling occurs. In some sparsely populated areas, a larger circle was used to increase the number of households. For three urban clinics (two in Kafue District and one in Chongwe District) a smaller circle was used, owing to overlap with the circle of other clinics. Each evaluation area is subdivided by overlaying a grid of approximately 900 x 900 meters (corresponding with 0.5 geographical minute of latitude). The number of households in each square is approximated using publically available satellite images (http://www.google.com/earth). If the number of households exceeds 50, the

Table 2 Study objectives, their indicators and data source

Objective	Indicator(s)	Primary data source
Reduce mortality	Age standardized mortality*	• Community survey
	Under 5 mortality	• Community survey
Improve coverage of child health services	Vaccine coverage	• Community survey
Improve coordination of key services to improve outcomes	Community HIV-1 viral load	• Community survey; DBS§ specimen
	Prevalence of uncontrolled hypertension	• Community survey;
	Prevalence of uncontrolled diabetes	• Community survey; DBS§ specimen
Implement a feasible and cost-effective intervention	Incremental cost-effectiveness of intervention	• Facility survey<br>• Medical record<br>• Community Survey

* Limited to individuals < 60 years of age

§ - Dried whole blood spot collected on filter paper

square is subsequently subdivided in four smaller squares. All squares with minimally five households are ranked in random order using a "rank probability proportionate-to-size" technique and are then visited in sequence, with all households in each successive grid approached until at least 120 households have been surveyed. In cases where no one is home to participate in the interview, we make up to three attempts to return, after which we count the household as unavailable. Each survey round has information from approximately 5040 households (42*120).

Survey instruments

The survey instrument was adapted from the Zambia Demographic and Health Survey (DHS) [15]. Team members begin by enumerating all adults and children who are usual members of household as well as visitors who spent the night prior to the enumeration day. The questionnaires focus on a wide range of issues, including household composition, resources, education of household members, recent death, sickness, health care utilization by household members, and other demographics. The questionnaire also seeks answers about disease knowledge, attitudes and perceptions, pregnancy, and child health. We created specific questionnaires for children, women, and men. We are using verbal autopsy [26,27] to better understand the circumstances surrounding reported deaths. Teams that include at least one registered nurse have been trained to administer the verbal autopsy surveys and may schedule separate visits to the home if necessary to gain access to key informants.

The surveys use an entire household enumeration method to identify all deaths occurring within the household in the year prior to the visit. For under-5 mortality, the household enumeration is being supplemented with a birth history method for estimating deaths. We are collecting height, weight, mid upper arm circumference (MUAC), and resting blood pressure on all participating adults, and height/length, weight, and MUAC on all participating children. We are also collecting a single dried blood spots (DBS) on filter paper for each participating adult and child.

The data and specimens we are collecting will allow us to survey several indicators of general adult health. These include 1) suppressed HIV disease – measured via assay of the DBS as a proportion of individuals in the population with HIV antibodies who have a suppressed viral load; 2) controlled blood pressure – measured as the proportion of hypertensive adults in the population who are normotensive at the time of household visit; and 3) controlled diabetes – measured via assay of DBS for glycated hemoglobin. Questionnaire data will be used to construct health care "coverage scores" for essential child and adult services.

Secondary objectives

The secondary objectives of the BHOMA intervention include a) improved coverage of key primary health interventions; b) improved overall coordination and effectiveness of the health system; and c) implementation of a feasibility and cost-effective intervention (Table 2). Our secondary objectives will be measured using a variety of methods. Health facility surveys, observations of patient-provider interaction, patient exit interviews and self-administered questionnaires are being implemented at health facility level to measure health system changes and also to provide data on inputs, processes, and outputs for causal chain analysis. Rapidly gathered qualitative data (focus group discussions, transect walks, and structured observations) in selected communities on key features of community types and local management of serious illness and death [28] are being used to assess social impact and the social context of the intervention, as well as to better understand findings from the community surveys and health facility studies.

Economic costing from a provider perspective is also under way. Collected data on costs will consist of the unit costs of 'fully' receiving the intervention and the

cost per change in age-standardized death rate and per death averted. The costs of delivering BHOMA services will be assessed using bottom-up, micro costing approach [29,30], which involves the quantification of all the resources required by the mode of delivery. Costs are being categorized into those borne by the health service and those by patients and their families. Both the recurrent and capital costs to the health service of training staff and delivering the intervention will be estimated.

Sample size and analysis

The sample size for our study was calculated using Hayes' formula for parallel cluster-randomized trials [31]. The estimate assumes an age-standardized mortality rate for those <60 years of 20/1000. Each of three cross-sectional surveys will recruit 150 households per cluster, of whom an estimated six members will be age <60 years. Each survey round will employ a 12-month "look back" period for the primary analysis. For a value of k between 0.2 and 0.3, we have > 90% power to detect a 35% reduction in mortality; for k=0.35, we have > 85% power to detect a 35% reduction. The trial will be analyzed using methods appropriate for a stepped wedge design cluster randomized trial [25]. In brief, this involves taking the mortality estimates from each sampled cluster and calculating a conditional probability for the events that occur during that time period being in the intervention or control clusters. We will then fit a generalized linear model and account for within-cluster correlation using a random effect.

Qualitative analyses

The project has a large qualitative research component to explore the effect of community beliefs and perceptions, traditional leadership, within-household power and permission structures, gender dynamics, and household resources on utilization of formal health care. Data are being obtained through focus group discussions and field interviews and then analyzed according to themes using the theoretical propositions case-study strategy [32] and framework-analysis [33] approach. Ongoing work around community beliefs and perceptions of death are already well under way and are being used to inform the conduct of our verbal autopsies. The qualitative team is also doing work around health care worker motivation. The learning generated from our qualitative analyses is periodically provided to the clinical teams to assist with their understanding of the project context. It is our hope that this learning will improve the QI teams' effectiveness over time.

Informed consent and ethical review

We obtain informed consent prior to administering any questionnaire, performing anthropometry, or drawing blood. In cases where the primary survey respondent cannot read or write, a non-biased literate witness joins the informed consent procedure to ensure objective provision of information is given about the study and that the patient understands the survey procedure. Ethical approval of the survey and accompanying intervention has been obtained from the institutional review boards at the University of Alabama at Birmingham (Birmingham, AL, USA), the University of North Carolina (Chapel Hill, NC, USA), the University of London (UK), and the University of Zambia Research Ethics Committee (Lusaka, ZM).

Discussion

The goal of our project is to measurably reduce mortality rates in a large, predominately rural population in Zambia. We aim to achieve this through a health systems intervention that puts delivery of high-quality, standardized, and monitored clinical care at its center. At this stage, the BHOMA team can report on key implementation successes and adaptations that have improved our approach to reducing mortality rates. (See Table 3 for a summary of the key successes, challenges and adaptations of the project observed thus far.) While the project is multifaceted, and involves community, facility, and district level activities, all our interventions are organized around the central idea that good clinical care is essential to good health outcomes. Equally important is community utilization of that care.

We realize, of course, that factors far beyond the scope of the health facility contribute to morbidity and mortality in rural Zambia. Underlying disease burden, water and sanitation, under-nutrition, and accidents are examples of factors that drive mortality, but that are largely outside the reach of the rural clinician. In addition, non-clinical public health interventions, such as childhood vaccine campaigns, bednet distribution, and nutritional programs also play a hugely important role in the public health.

The BHOMA intervention does not attempt to fix everything. We have chosen to focus upon the delivery of good clinical services and are attempting to demonstrate that "if you build it, they will come." Once community members begin to realize that diagnostics and drugs are available at their local facility and that their local clinicians can competently use them, they will present earlier and more often, keep their follow-up appointments, and adhere to prescribed treatment. This virtuous cycle will be encouraged through the use of CHWs.

A major strength of our project lies in the rigor of the evaluation we have planned. Since our intervention is being implemented in six facilities (and each facility's catchment area) at a time, this allows for a comparative trial. The intervention is being implemented at randomly selected facilities in a stepped wedge [24] design. At the end of the implementation, we will be able to measure mortality and various other outcomes and attribute

Table 3 Zambia PHIT Implementation progress: success, challenges, adaptations

Successes
**Local Ownership**<br>**The top-level leadership from each participating health district has been involved in the BHOMA project since its conception (including the application for initial funding) and each district has provided substantial human resources to program implementation.**
**Collaboration and partnership**<br>**We have successfully leveraged the substantial infrastructure and resources available through other programs sponsored by our group (e.g., support for HIV/AIDS care and treatment services) in the target districts. This has fostered trust and familiarity between project teams and regular Ministry of Health providers at the implementation sites.**
**Effective use of existing technology**<br>**Mobile phone coverage is now nearly 100% in Zambia. Our project was forward thinking in its adoption of this relatively low-cost technology for managing community health worker outreach activities.**
Challenges
Adaptations
**Monitoring CHW performance with a Lot Quality Assurance System (LQAS) methodology**<br>**To ensure the community health workers were actually performing the task we charged them with, we introduced LQAS sampling of households. Most CHWs are performing very well, but those who were not can now be identified and remediated.**
**Incentives for Traditional Birth Attendants (TBAs)**<br>**We noticed low rates of institutional delivery among women in rural areas and that many of these women were using the services of TBAs. We co-opted the TBAs into our community program, providing incentive payments for early referral of pregnant mothers to antenatal care, institutional delivery, and home, postnatal visitations. TBAs are now being monitored through the same common performance indicator system used for clinical care providers.**
**Neonatal follow up**<br>**During implementation it became apparent that the form used to capture a child's sick visit was inadequate to guide the special care required for sick neonates. Therefore, the project went through an extensive consultative process to develop and implement a new neonatal form.**

changes in those metrics (if any) to the intervention. This is an unusual strength for such a large, complex health systems intervention. In addition, the electronic system we have implemented for patient-level monitoring will allow us to precisely quantify whether our intervention has the effect of improving health-seeking behavior in the community. Patients can be tracked seamlessly between the facility and the community and their service utilization quantified over time.

The project also faces several risks. By investing so heavily into clinical service delivery, we are in a sense gambling that our efforts to get patients to come in for care will work. If improved quality does not translate into better service utilization, then the effect of our intervention will be mitigated. Put simply, a facility-based intervention cannot help those who don't come in for care. We are, to some extent, constrained by current policy around the use of community health volunteers in Zambia. Unlike some neighboring countries, Zambia has extended only limited diagnostic and prescriptive responsibilities to lay health workers (they do not start antibiotics for childhood pneumonia, for instance). Our intervention uses CHWs as extenders of the facility-based care who are charged with continuous monitoring of their respective catchment areas, making referrals, and following up on patients who miss their appointments. Finally, it may not be realistic to expect that our clinical focus will bring the same immediate success in primary health care as we've seen in other services, like HIV care [34,35]: first, it is easier to establish a new practice correctly than to change existing practice; and second, primary health care is a more diffuse target, with a wider range of protocols, equipment, and drugs needed to deliver good care.

The patient-provider interaction is a critical interface where the community and the health system meet. Our project will invest in this interaction to improve population health. We hope to maximize learning from this project through multiple levels of data collection and program documentation. If we demonstrate success, we are hopeful that the model could be applied widely throughout Zambia and elsewhere.

List of abbreviations used

ANC: Antenatal Care; BHOMA: Better Health Outcomes through Mentoring and Assessment; CHW: Community health worker; DHOs: District health offices; DILSAT: District Integrated Logistics and Supplies Assessment Tool; DBS: Dried blood spots; DHS: Demographic health survey; EmONC: Emergency obstetrical and newborn care; HMIS: Health management information system; IMAI: Integrated management of adult illnesses; IMCI: Integrated management of childhood illnesses; MCH: Maternal child health; MUAC: Mid-upper arm circumference; MDGs: Millennium Development Goals; MoH: Ministry of Health; NHCs: Neighborhood health committees; QI: Quality improvement; TBA: Traditional birth attendant.

Competing interests

The authors declare that they have no competing interests.

Acknowledgements
This work is supported by the African Health Initiative of the Doris Duke Charitable Foundation (Grant # 2009060).

Declarations
This article has been published as part of *BMC Health Services Research* Volume 13 Supplement 2, 2013: Improving primary health care to achieve population impact: the African Health Initiative. The full contents of the supplement are available online at http://www.biomedcentral.com/bmchealthservres/supplements/13/S2. Publication of this supplement was supported by the African Health Initiative of the Doris Duke Charitable Foundation.

Author details
[1]Department of Obstetrics and Gynecology, University of North Carolina School of Medicine, Chapel Hill, NC, USA. [2]Primary Care and Health Systems Department, Centre for Infectious Disease Research, Lusaka, Zambia. [3]Development Department, Centre for Infectious Disease Research, Lusaka, Zambia. [4]ZAMBART PROJECT, Zambia AIDS Related Tuberculosis Project, Lusaka, Zambia. [5]School of Public Health, University of Alabama at Birmingham, Birmingham, AL, USA.

Published: 31 May 2013

References

1. International Development Association: **Country Assistance Strategy Progress Report for the Republic of Zambia for the Period 2008-2012.** 2011.
2. WHO: **Integrated Management of Childhood Illnesses.** Geneva: WHO; 2008.
3. World Bank: **World Development Indicators.** 2010, Accessed 4 September 2012 at [http://data.worldbank.org/country/zambia].
4. Adedimeji A, Malokota O, Manafa O: **Implementing antiretroviral therapy programs in resource-constrained settings: lessons from Monze, Zambia.** *Journal of Public Health Policy* 2011, **32(2)**:198-210.
5. Brugha R, *et al*: **Health workforce responses to global health initiatives funding: a comparison of Malawi and Zambia.** *Human Resources for Health* 2010, **8**:19.
6. Kruse GR, *et al*: **Burnout and use of HIV services among health care workers in Lusaka District, Zambia: a cross-sectional study.** *Human Resources for Health* 2009, **7**:55.
7. Stringer EM, *et al*: **A randomized trial of the intrauterine contraceptive device vs hormonal contraception in women who are infected with the human immunodeficiency virus.** *American Journal of Obstetrics and Gynecology* 2007, **197(2)**:144 e1-8.
8. Sundewall J, *et al*: **The Paris Declaration in practice: challenges of health sector aid coordination at the district level in Zambia.** *Health Research Policy and Systems/BioMed Central Health Services Research* 2009, **7**:14.
9. WHO: **The World Health Report 2006: Working Together for Health.** Geneva: WHO; 2006.
10. Dayrit MM, Dolea C, Dreesch N: **Addressing the Human Resources for Health crisis in countries: How far have we gone? What can we expect to achieve by 2015?** *Revista peruana de medicina experimental y salud publica* 2011, **28(2)**:327-36.
11. Walsh A, *et al*: **Task sharing in Zambia: HIV service scale-up compounds the human resource crisis.** *BioMed Central Health Services Research* 2010, **10**:272.
12. Ministry of Finance and National Planning, UNDP: **Millennium Development Goals Progress Report.** Lusaka: UNDP; 2011.
13. UNICEF: **The State of the World's Children.** New York: UNICEF; 2012.
14. WHO, UNFPA, World Bank: **Trends in Maternal Mortality: 1990 to 2010.** Geneva: WHO; 2012.
15. Central Statistical Office, Ministry of Health, Tropical Disease Research Centre, University of Zambia, Macro International Inc.: **Zambia Demographic and Health Survey 2007.** Calverton, MD: Macro International; 2009.
16. WHO: **Integrated Management of Adult Illness: Acute Care.** Geneva: WHO; 2004.
17. WHO: **Integrated Management of Adult Illness: Chronic HIV Care with ARV Therapy and Prevention.** Geneva: WHO; 2004.
18. WHO: **Integrated Management of Adult Illness: General Principles of Good Chronic Care.** Geneva: WHO; 2004.
19. WHO: **Integrated Management of Adult Illness: Palliative Care.** Geneva: WHO; 2004.
20. WHO: **Operations Manual for Delivery of HIV Prevention, Care and Treatment at Primary Health Centres in High-Prevalence, Resource-constrained Settings.** Geneva: WHO; 2008.
21. Ministry of Health: **Emergency Obstetric and Newborn Care (EmONC) Guide.** Lusaka: Ministry of Health; 2011.
22. Central Statistical Office: **Census of Population and Housing Preliminary Population Figures.** Lusaka: Central Statistical Office; 2010.
23. Morris MB, *et al*: **Use of task-shifting to rapidly scale-up HIV treatment services: experiences from Lusaka, Zambia.** *BioMed Central Health Services Research* 2009, **9**:5.
24. Brown CA, Lilford RJ: **The stepped wedge trial design: a systematic review.** *BMC Medical Research Methodology* 2006, **6**:54.
25. **Cluster randomised trials.** In *Interdisciplinary Statistics Series.* Boca Raton: Chapman and Hall;Hayes R, Moulton LH, Keiding N, et al 2009.
26. Mudenda SS, *et al*: **Feasibility of using a World Health Organization-standard methodology for Sample Vital Registration with Verbal Autopsy (SAVVY) to report leading causes of death in Zambia: results of a pilot in four provinces, 2010.** *Population Health Metrics* 2011, **9**:40.
27. Turnbull E, *et al*: **Causes of stillbirth, neonatal death and early childhood death in rural Zambia by verbal autopsy assessments.** *Tropical Medicine & International Health* 2011, **16(7)**:894-901.
28. Bond V: **Terrains and tuberculosis: the model applied in urgent public health settings.** In *The Capability of Places: Methods for Modelling Community Response to Intrusion and Change.* UK: Pluto Press;Wallman S (Ed) in association with Bond V, Lo Conte R, Montuori A, Vidali M 2011:80-110.
29. Batwala V, *et al*: **Cost-effectiveness of malaria microscopy and rapid diagnostic tests versus presumptive diagnosis: implications for malaria control in Uganda.** *Malaria Journal* 2011, **10**:372.
30. Drake TL, *et al*: **Cost analysis of school-based intermittent screening and treatment of malaria in Kenya.** *Malaria Journal* 2011, **10**:273.
31. Hayes RJ, Bennett S: **Simple sample size calculation for cluster-randomized trials.** *International Journal of Epidemiology* 1999, **28(2)**:319-26.
32. Yin RK: **Case Study Research Design and Methods.** Los Angeles: SAGE Publications; 2009.
33. Green J, Thornogood N: **Qualitative Methods for Health Research.** London: SAGE Publications; 2009.
34. Stringer JS, *et al*: **Rapid scale-up of antiretroviral therapy at primary care sites in Zambia: feasibility and early outcomes.** *JAMA* 2006, **296(7)**:782-93.
35. Boton-Moore C, *et al*: **Clinical outcomes and CD4 cell response in children receiving antiretroviral therapy at primary health care facilities in Zambia.** *JAMA* 2007, **298**:1888-99.

doi:10.1186/1472-6963-13-S2-S7
Cite this article as: Stringer *et al.*: Protocol-driven primary care and community linkages to improve population health in rural Zambia: the Better Health Outcomes through Mentoring and Assessment (BHOMA) project. *BMC Health Services Research* 2013 **13**(Suppl 2):S7.

Submit your next manuscript to BioMed Central and take full advantage of:

- **Convenient online submission**
- **Thorough peer review**
- **No space constraints or color figure charges**
- **Immediate publication on acceptance**
- **Inclusion in PubMed, CAS, Scopus and Google Scholar**
- **Research which is freely available for redistribution**

Submit your manuscript at
www.biomedcentral.com/submit

BioMed Central
